# Clinical Surveillance Technologies in Nonintensive Care Unit Hospital Settings: Systematic Review and Bayesian Network Meta-Analysis of Randomized Trials

**DOI:** 10.2196/98205

**Published:** 2026-09-24

**Authors:** Xinbo Yin, Xiangmin Li, Guoqing Huang, Xiaokai Wang

**Affiliations:** 1 Teaching and Research Section of Clinical Nursing, Xiangya Hospital, Central South University, Changsha, Hunan, China; 2 Department of Emergency Medicine, Xiangya Hospital, Central South University, 87 Xiangya Road, Changsha, Hunan, 410008, China, 86 13873152091; 3 National Clinical Research Center for Geriatric Diseases (Xiangya Hospital), Central South University, Changsha, Hunan, China

**Keywords:** clinical deterioration, early warning scores, machine learning, continuous physiologic monitoring, Bayesian network meta-analysis, hospitalized patients, intensive care unit transfer, trial sequential analysis

## Abstract

**Background:**

Failure to recognize clinical deterioration in hospitalized patients has prompted the development of rule-based electronic surveillance (RB-ES), predictive model–based electronic surveillance (PM-ES), and continuous physiologic monitoring (CPM). However, their comparative effects on patient-centered outcomes remain uncertain.

**Objective:**

The aim of this study is to compare the clinical effects of emergent intensive RB-ES, PM-ES, CPM, and local standard care in nonintensive care unit hospital settings.

**Methods:**

We searched PubMed, Embase via online, the Cochrane Central Register of Controlled Trials via the Cochrane Library, and the Web of Science Core Collection from inception through February 28, 2026. Targeted supplementary surveillance, including trial-registry follow-up, backward and forward citation tracking, and known-item searches for newly available reports of registered trials, continued through July 22, 2026. Randomized, cluster-randomized, randomized crossover, and stepped-wedge trials were eligible. Interventions were classified according to their principal randomized function rather than their commercial or algorithmic labels. The primary outcomes were all-cause in-hospital or up-to-30-day mortality, and unplanned or emergent intensive care unit transfers. Bayesian random-effects network meta-analyses were performed using study-level adjusted relative effects. Secondary outcomes were evaluated using construct-specific pairwise meta-analyses. Risk of bias was assessed using the appropriate RoB 2 tool, and confidence in the network estimates was manually evaluated using the CINeMA (Confidence in Network Meta-Analysis) framework. Trial sequential analysis was retained as an exploratory supplementary analysis.

**Results:**

The review included 28 independent trials. Nine met strict digital-surveillance criteria; 7 contributed to at least 1 network, while 2 contributed only to sensitivity analyses because of outcome-definition or zero-event limitations. Six trials with 13,716 observations contributed to the mortality network. Compared with standard care, odds ratios were 0.91 (95% credible interval [CrI] 0.35‐2.30) for RB-ES, 1.22 (95% CrI 0.61‐2.31) for PM-ES, and 0.70 (95% CrI 0.36‐1.29) for CPM. Six trials with 13,441 observations contributed to the intensive care unit transfer network. Corresponding odds ratios were 1.20 (95% CrI 0.56‐2.59), 0.95 (95% CrI 0.58‐1.52), and 0.70 (95% CrI 0.37‐1.28), respectively. Expanded rule-based surveillance-response systems did not clearly reduce cardiac arrest or cardiopulmonary resuscitation (OR 0.94, 95% CI 0.77‐1.14). CPM (ratio of means 0.91, 95% CI 0.76‐1.09) and predictive model–based surveillance (ratio of means 0.99, 95% CI 0.47‐2.08) showed no clear effect on hospital length of stay. Trial sequential analyses were inconclusive. Confidence in all network comparisons was very low.

**Conclusions:**

Current randomized evidence does not establish a reliable clinical-effectiveness hierarchy among RB-ES, PM-ES, and CPM. Continuous monitoring showed directionally favorable but imprecise estimates for several outcomes. Surveillance strategies should therefore not be selected solely according to algorithm class or predictive complexity; their clinical effects may also depend on the target population, background monitoring, workflow integration, alert presentation, and clinical response pathway.

## Introduction

Failure to recognize and respond to clinical deterioration in hospitalized patients remains an important source of potentially preventable morbidity and mortality. Physiological abnormalities, including tachycardia, hypotension, tachypnea, hypoxemia, and altered consciousness, can occur for several hours before cardiac arrest, unplanned transfer to an intensive care unit (ICU), or death. This interval may provide an opportunity for earlier assessment and treatment when deterioration is reliably detected and communicated [1].

Clinical surveillance is not a single diagnostic test. It is a multicomponent care process that includes acquisition of patient data, interpretation of those data, communication of risk, and delivery of an appropriate clinical response. Its effectiveness may therefore depend on the complete surveillance-response pathway rather than on the accuracy of the detection algorithm alone [2,3].

Three broad technological approaches are commonly used in non-ICU hospital settings. Rule-based electronic surveillance (RB-ES) applies prespecified deterministic criteria or aggregate scores to intermittently collected clinical data and generates an alert or escalation prompt. Predictive model–based electronic surveillance (PM-ES) uses empirically fitted statistical or machine-learning models to estimate patient-specific deterioration risk and communicates that risk through an alert or visual display. Continuous physiologic monitoring (CPM) acquires physiologic data continuously or near-continuously and makes those data available to clinicians through displays or automated alerts.

These categories describe the principal randomized function of an intervention rather than its commercial label. Individual systems may additionally incorporate clinical education, workflow redesign, pharmacist review, or rapid-response-team involvement. Consequently, systems with similar underlying algorithms may have different clinical effects when their alert delivery and response pathways differ. Conversely, systems based on different algorithms may produce similar effects when implemented through the same clinical workflow.

Previous evidence syntheses have generally assessed early warning scores (EWSs), predictive alerts, and continuous monitoring separately. They also include a large number of retrospective model-development studies and nonrandomized implementation studies, which do not establish whether the implementation of a surveillance strategy improves patient outcomes [4-6].

Comparative interpretation is further complicated by variation in clinical settings, baseline risk, target conditions, background monitoring, alert presentations, outcome definitions, and responsibility for clinical response. Network meta-analysis (NMA) can compare multiple intervention classes through direct and indirect evidence, but its validity depends on clinically defensible intervention nodes and a plausible transitivity assumption. Treatment rankings can be particularly misleading when the network is sparse, when active interventions have not been compared head-to-head, and when intervention definitions are heterogeneous.

We therefore conducted an updated systematic review and a Bayesian NMA of randomized trials evaluating clinical surveillance technologies in non-ICU hospital settings. The primary objective was to compare RB-ES, PM-ES, CPM, and local standard care (SC) for short-term mortality and unplanned or emergent ICU transfer. Secondary objectives were to evaluate confirmed cardiac arrest or cardiopulmonary resuscitation, hospital length of stay (LOS), and construct-specific treatment-activation outcomes and to characterize the certainty, contribution structure, and limitations of the available randomized evidence.

## Methods

### Study Design and Reporting

This systematic review and Bayesian NMA was conducted in accordance with PRISMA 2020 (Preferred Reporting Items for Systematic Reviews and Meta-Analyses; Checklist 1), the PRISMA-S (Preferred Reporting Items for Systematic Reviews and Meta-Analyses Literature Search Extension), and the Cochrane Handbook for Systematic Reviews of Interventions [7-10].

This review was registered in PROSPERO (International Prospective Register of Systematic Reviews) on April 6, 2026 (CRD420261356381). Registration occurred after the review had commenced and after formal searching and screening had begun, but before data extraction, risk-of-bias assessment, and data synthesis. Deviations from the registered methods and reviewer-requested analytical amendments are documented in Multimedia Appendix 1.

### Eligibility Criteria

Eligible populations received care in emergency departments, acute admission or observation units, general medical or surgical wards, stroke units, or step-down units. Studies conducted exclusively in ICUs were excluded. Mixed setting trials were retained when the intervention was implemented in an eligible non-ICU setting and at least 1 relevant outcome was attributable to non-ICU care.

We included individually randomized, cluster-randomized, randomized crossover, and stepped-wedge cluster-randomized trials. Pilot and feasibility trials were eligible, but these were distinguished from definitive effectiveness trials.

Eligible interventions were surveillance strategies intended to identify or communicate the risk of general clinical deterioration, sepsis, physiologic instability, or related adverse clinical events. Studies were excluded if the randomized intervention principally involved procedural sedation monitoring, monitoring used to guide fluid or hemodynamic treatment, one-time triage, long-term prognostic risk stratification performed after the completion of clinical assessment, or disease-specific multicomponent clinical decision support from which the independent contribution of deterioration surveillance could not be isolated.

Eligible comparators included local SC, a silent or hidden alert, intermittent vital-sign monitoring, or an existing early warning strategy applied equally to both randomized groups.

The primary outcomes were all-cause in-hospital mortality or mortality reported within 30 days, and unplanned or emergent transfer from an eligible non-ICU unit to an ICU. Longer mortality follow-up and broader definitions of ICU admission were examined in sensitivity analyses.

Secondary outcomes included confirmed cardiac arrest or cardiopulmonary resuscitation, hospital LOS, and treatment-activation outcomes. Code Blue activation, rapid response team activation, and cardiac arrest team calls were not treated as equivalent to a confirmed cardiac arrest.

### Information Sources and Search Strategy

We searched PubMed (National Center for Biotechnology Information), Embase via online (Elsevier) [11], the Cochrane Central Register of Controlled Trials via the Cochrane Library, and the Web of Science Core Collection via Clarivate from database inception through February 28, 2026. No language restrictions were applied.

To identify reports becoming available after the database-search cutoff and to link multiple reports of the same trials, we conducted targeted supplementary surveillance from March 1 through July 22, 2026. This process included searches of National Library of Medicine via online [12] and the World Health Organization (WHO) International Clinical Trials Registry Platform, backward and forward citation tracking of eligible trials and relevant reviews, and known-item searches for newly published reports of completed registered trials. Fourteen additional primary randomized trial reports were added to the final review library through backward and forward citation tracking and targeted supplementary surveillance. A report-level identification log is provided in Table S17 in Multimedia Appendix 1. Protocols, trial registrations, statistical analysis plans, secondary reports, corrections, and online supplements used only for trial linkage, reporting-bias assessment, or correction of extracted estimates were documented separately and were not counted as independent trials.

We additionally searched National Library of Medicine via online [12] and the WHO International Clinical Trials Registry Platform, reviewed reference lists of eligible trials, and relevant systematic reviews, and performed backward and forward citation tracking. Trial registrations, protocols, statistical analysis plans, secondary reports, and corrections were linked to their underlying trials.

### Study Selection and Data Extraction

After duplicate removal using EndNote 21 (Clarivate Analytics), 2 reviewers independently screened titles and abstracts and subsequently assessed full-text reports. Screening decisions were recorded in Microsoft Excel (Microsoft 365, Microsoft Corp). Disagreements were resolved through discussion or consultation with a third reviewer.

The original item-level screening records did not permit a valid retrospective calculation of Cohen κ. The unsupported statement in the original paper that κ had been assessed was therefore removed.

Data were extracted into a standardized Microsoft Excel spreadsheet (Microsoft 365) and were independently verified by a second reviewer. Extracted information included trial design, randomization unit, analysis unit, clinical setting, eligibility criteria, intervention components, comparator, background monitoring, alert presentation, response pathway, outcome definition, follow-up period, reported effect measure, and whether the estimate accounted for clustering or calendar period.

When continuous outcomes were reported only as medians and IQRs, means and SDs were estimated using established methods and were analyzed only as exploratory sensitivity evidence [13,14].

### Trial-Report Linkage and Data Units

Before the quantitative synthesis, all publications were linked to their underlying independent trials. A secondary report was not counted as an independent trial and was not permitted to contribute the same participants twice within a single outcome analysis.

The reports by Dean et al [15] and Carr et al [16] were linked to the same electronic pneumonia clinical decision-support trial and excluded from the surveillance network because the intervention was a disease-specific multicomponent decision-support system.

The reports from the Haegdorens trial, the Weenk trial, the SCREEN trial protocol and statistical analysis plan, and the correction to the CONCERN trial were similarly linked under common trial identifiers [17-23].

We distinguished unique participants from number of admissions, hospital encounters, and inpatient visits. Because the unit of analysis differed across trials, a single overall participant total was not calculated. Outcome-specific analyzable denominators were reported instead.

### Intervention Classification and Node Assignment

Interventions were classified according to the principal randomized difference between study groups, rather than commercial terminology.

RB-ES comprised electronic systems that applied prespecified deterministic criteria or aggregate scores to intermittently collected clinical data and generated an alert or escalation prompt.

PM-ES comprised systems that generated patient-specific deterioration risk estimates using empirically fitted statistical or machine-learning models and communicated those estimates through alerts or visual displays.

CPM comprised the continuous or near-continuous acquisition of physiologic data, which were made available through displays or alerts when access to continuous monitoring constituted the principal randomized contrast.

Local SC was defined as the background monitoring strategy applied equally to both groups and could include intermittent vital-sign assessment or an existing EWS.

Expanded rule-based surveillance-response interventions, passive monitoring, disease-specific monitoring, mixed emergency department–inpatient settings, and multicomponent interventions were retained for sensitivity or narrative analyses rather than combined indiscriminately within the strict network.

Two reviewers independently confirmed node assignments without reference to study results. The node definitions, study-level assignments, and rationales are provided in Multimedia Appendix 2.

### Transitivity Assessment

Transitivity was evaluated by comparing clinical settings, baseline risk, target conditions, enrollment strategies, background monitoring, alert presentations, response pathways, and outcome time horizon across direct comparisons.

The strict networks were limited to more clinically comparable digital-surveillance interventions. Nevertheless, active-vs-active comparisons were interpreted cautiously because relevant effect modifiers remained unevenly distributed across nodes.

### Risk of Bias

Two reviewers independently assessed each eligible outcome result using RoB 2 for individually randomized trials and the corresponding cluster-randomized or crossover versions, when appropriate. Assessments targeted the effect of assignment to the intervention. Domain-level and overall judgments were classified as low risk, some concerns, or high risk [24].

### Effect-Measure Selection and Harmonization

Study-level effect estimates adjusted for cluster randomization, crossover period, calendar time, and other prespecified design features were prioritized.

Odds ratios (ORs) were used for the primary binary-outcome networks. Adjusted risk ratios (RRs) were converted to ORs using the reported control-group risk, with sensitivity analyses conducted on the original risk-ratio scale. Hazard ratios were not treated as equivalent to ORs and were retained for separate sensitivity or narrative analyses.

Hospital LOS and treatment-time outcomes were analyzed using ratios of arithmetic means rather than standardized mean differences. Adjusted incidence rate ratios and time-to-event estimates that could not be harmonized with arithmetic mean ratios were reported separately.

### Bayesian NMA

A contrast-based Bayesian NMA was performed using JAGS (Just Another Gibbs Sampler; JAGS Project, SourceForge) through the gemtc package in R version 4.4.1 (R Foundation for Statistical Computing). Study-level log ORs and SEs were used as inputs [25].

Random-effects models were prespecified as the primary models because clinical heterogeneity was anticipated. A common heterogeneity parameter was assumed across comparisons, using a half-normal prior distribution with a scale of 0.5. Sensitivity analyses used scale parameters of 0.2 and 1.0. Fixed-effect models were examined only as sensitivity analyses and were not selected based on the deviance information criterion alone.

Four Markov chain Monte Carlo chains were run with 20,000 adaptation iterations followed by 100,000 sampling iterations per chain; every 10th iteration was retained. Convergence was assessed using trace and density plots, the Brooks-Gelman-Rubin statistic, effective sample sizes, and Geweke diagnostics. R-hat values below 1.05 were considered acceptable.

Treatment rankings and surface under the cumulative ranking curve values were not used as principal results because the strict networks were sparse, active strategies had not been compared directly, and rankings could imply an unsupported clinical hierarchy.

### Network Geometry and Contribution Analysis

Direct-comparison and study-level contributions to each network estimate were quantified using a flow-decomposition approach. Study-level contributions within each direct comparison were allocated according to random-effects inverse-variance weights [26].

Because the strict networks were star-shaped and contained no closed loops, node splitting and statistical testing of incoherence were not possible. This was reported as an inability to assess incoherence rather than as evidence that incoherence was absent.

### Secondary Meta-Analyses

Construct-specific pairwise random-effects meta-analyses were conducted using restricted maximum likelihood estimation and Hartung-Knapp CIs. Classic random-effects intervals were examined in sensitivity analyses.

Confirmed cardiac arrest or cardiopulmonary resuscitation, Code Blue activation, rapid response team activation, hospital LOS, and differently anchored treatment-activation outcomes were analyzed separately.

### Sensitivity Analyses

Mortality sensitivity analyses included an extended follow-up definition incorporating in-hospital mortality occurring within 90 days, inclusion of a zero-event trial using a continuity correction, exclusion of reconstructed Downing event counts, and alternative half-normal heterogeneity priors with scale parameters of 0.2 and 1.0.

ICU-transfer sensitivity analyses included a broader definition of subsequent ICU admission, the inclusion of a zero-event trial, and alternative heterogeneity priors.

Additional expanded analyses examined emergency department predictive alerts, passive continuous monitoring, disease-specific monitoring, and multicomponent surveillance-response interventions.

### Trial Sequential Analysis

Exploratory, retrospective, trial sequential analyses were conducted for sufficiently comparable direct CPM comparisons. Analyses used RRs with Mantel-Haenszel weighting, a 2-sided α value of .05, 80% power, an anticipated relative risk reduction of 20%, Lan-DeMets O’Brien-Fleming alpha-spending boundaries, no futility boundary, DerSimonian-Laird random-effects estimation with the Hartung-Knapp-Sidik-Jonkman adjustment, diversity-adjusted required information sizes, and a continuity correction of 0.5 for zero cells [27].

Trial sequential analysis was used only to assess information size and was not used as a substitute for certainty-of-evidence assessment.

### Risk of Bias Due to Missing Evidence

We searched trial registries and linked trial registrations, protocols, statistical analysis plans, corrections, and secondary reports to assess selective nonpublication and selective outcome availability.

Comparison-adjusted funnel plots were generated descriptively for the primary mortality and ICU-transfer networks. Formal tests of funnel-plot asymmetry and trim-and-fill analyses were not performed because fewer than 10 trials contributed to each primary network [28]. Across-studies bias was evaluated manually within CINeMA (Confidence in Network Meta-Analysis).

### Certainty of Evidence

Confidence in network estimates was evaluated manually using CINeMA across 6 domains: within-study bias, reporting bias, indirectness, imprecision, heterogeneity, and incoherence. ORs between 0.80 and 1.25 were prespecified as indicating no clinically important difference. Confidence was classified as high, moderate, low, or very low [29].

Because the primary networks contained no closed loops, incoherence was classified as not statistically assessable.

### Ethical Considerations

This systematic review used aggregate data from previously published studies and did not involve identifiable individual-level participant data. Additional institutional ethics approval and informed consent were therefore not required.

## Results

### Study Selection

Database searching identified 1645 records, and backward and forward citation tracking, together with updated supplementary searching, identified 14 additional reports. After the removal of 582 duplicate database records, 1063 titles and abstracts were screened and 968 were excluded.

Ninety-five reports identified through database searching and 14 reports identified through other methods were assessed in full text. Eighty-one reports were excluded because of an ineligible intervention or surveillance purpose (n=35), ineligible study design (n=22), ineligible population or setting (n=15), or absence of an eligible patient-centered outcome or sufficient outcome data (n=9).

**Figure 1.**
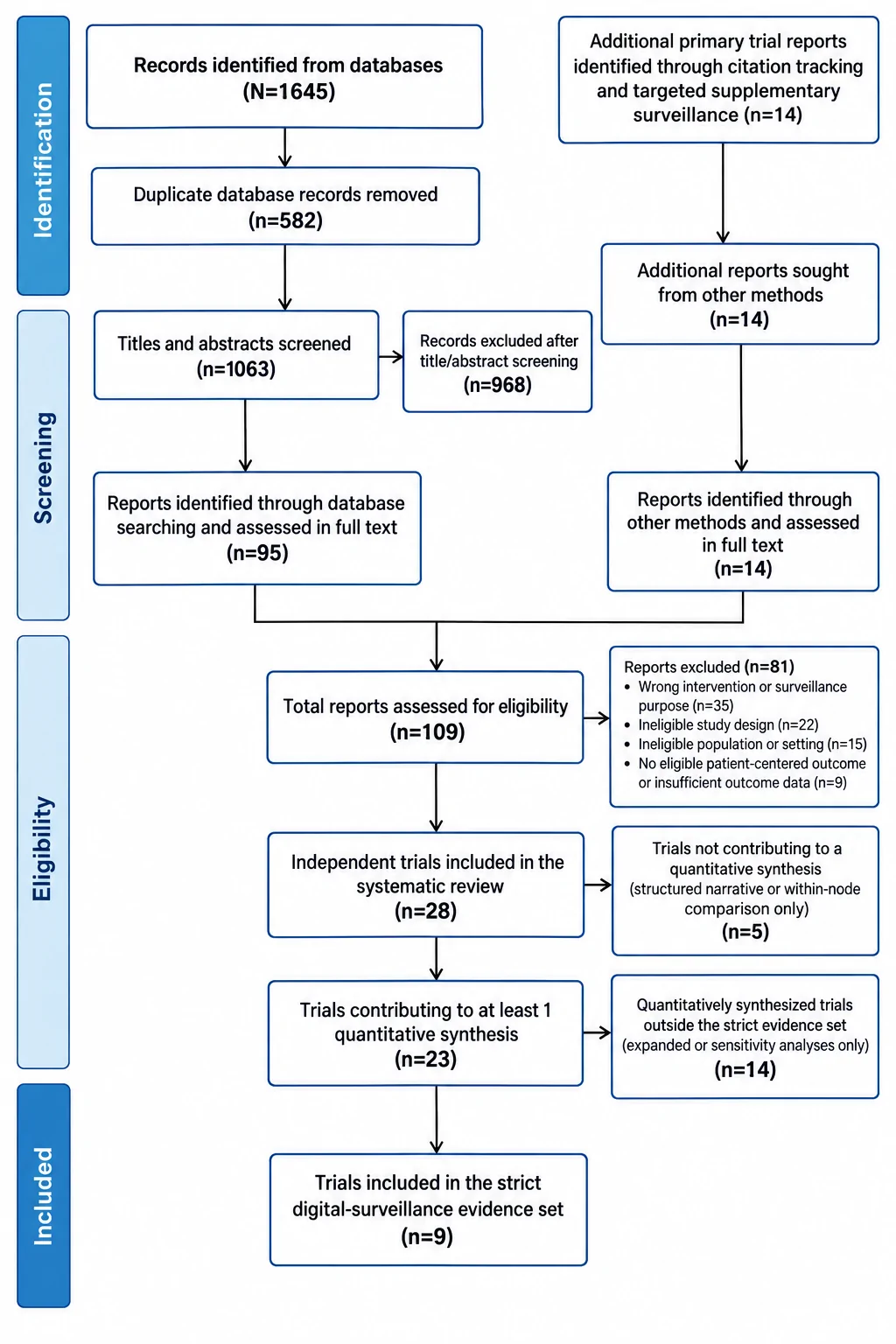
PRISMA (Preferred Reporting Items for Systematic Reviews and Meta-Analyses) 2020 flow diagram. Database records and reports identified through supplementary methods are displayed as separate pathways. Twenty-eight independent trials were included in the systematic review; 23 contributed to at least 1 quantitative synthesis and 9 contributed to the strict digital-surveillance evidence set.

Twenty-eight independent trials were included in the systematic review. Twenty-three trials contributed to at least 1 quantitative synthesis, and 9 formed the strict digital-surveillance evidence set (Figure 1).

### Study Characteristics and Revised Classification

The 28 included trials evaluated strict electronic surveillance, expanded surveillance-response systems, continuous monitoring, or within-node implementation variants.

Nine trials met the strict digital-surveillance eligibility criteria. Seven contributed to at least 1 strict primary network. SCREEN contributed only to the extended follow-up mortality and broader ICU-admission sensitivity analyses, whereas Paul contributed only to the zero-event ICU-transfer sensitivity analysis and exploratory trial sequential analysis (TSA) [30-38].

Fourteen additional trials were retained in expanded quantitative sensitivity analyses because of broader surveillance-response components, mixed settings, disease-specific populations, passive monitoring, or methodological limitations [17,39-51].

Five trials were retained for structured narrative synthesis or within-node comparisons [52-56].

The clinical settings included emergency departments, acute admission units, general medical and surgical wards, postoperative wards, stroke units, and mixed acute-care units. Analysis units included participants, admissions, hospital encounters, and inpatient visits. Trial characteristics, classifications, and synthesis roles are summarized in Multimedia Appendix 2.

### Risk of Bias

**Figure 2.**
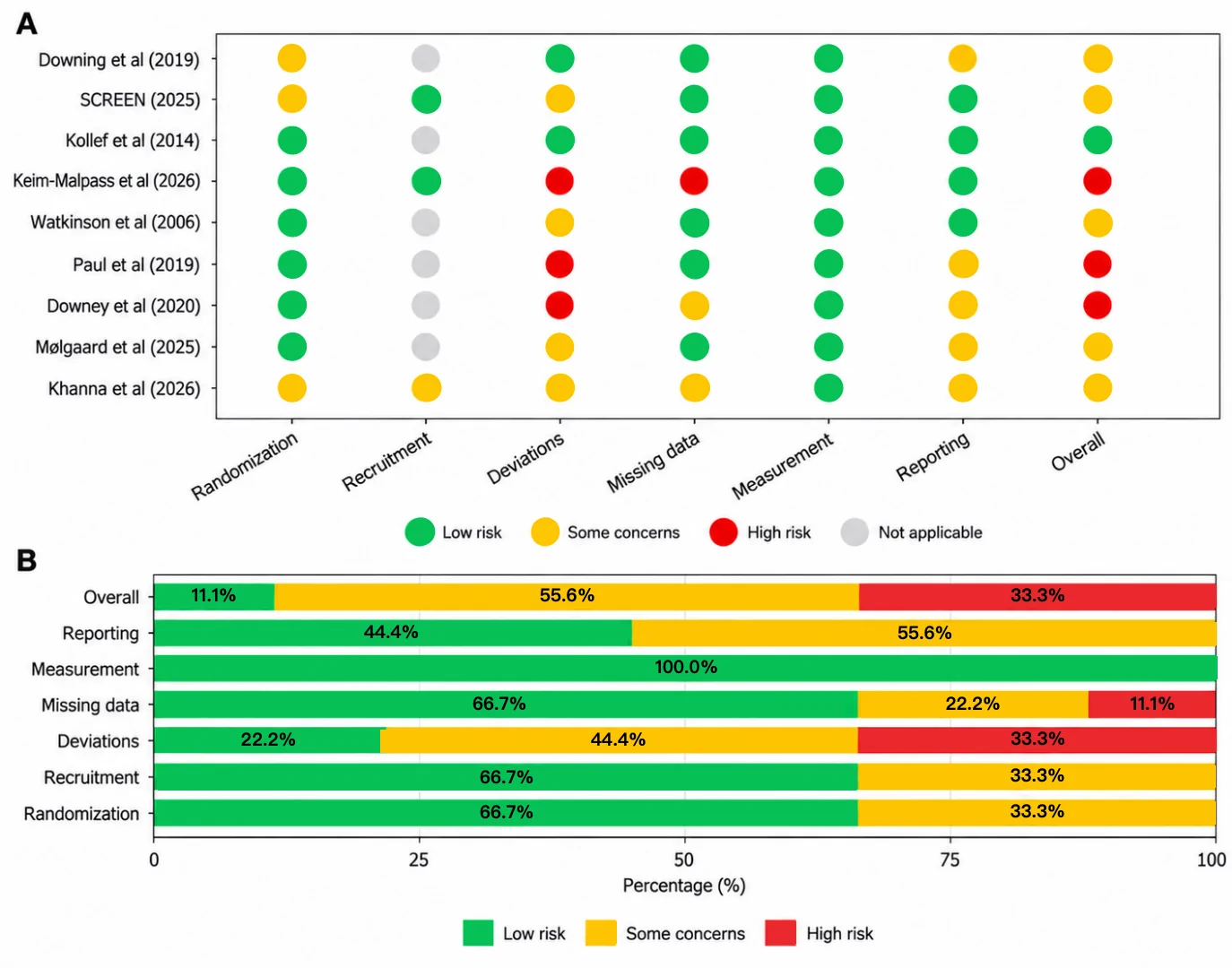
Risk-of-bias assessment for the 9 trials in the strict digital-surveillance evidence set. (A) Outcome-level RoB 2 judgments. (B) The proportion of judgments classified as low risk, some concerns, or high risk across domains. Domain D1b applies only to cluster-randomized and crossover trials [30-38].

Among the 9 trials in the strict digital-surveillance evidence set, 1 was judged to have low overall risk of bias, 5 raised some concerns, and 3 were at high risk (Figure 2 [30-38]).

The principal concerns were intervention nonadherence, postrandomization patient movement, incomplete exposure to continuous monitoring, selective censoring, and selective availability of patient-centered outcomes in pilot or feasibility trials.

The CoMET trial was judged to be at high risk because clinicians preferentially transferred patients perceived to be sicker to display-on beds, and bed movement resulted in censoring [33]. The VIGILANCE and TRaCINg pilot trials were judged to be at high risk because of substantial intervention nonadherence, monitoring discontinuation, or crossover [34,35].

### Network Geometry and Evidence Contributions

**Figure 3.**
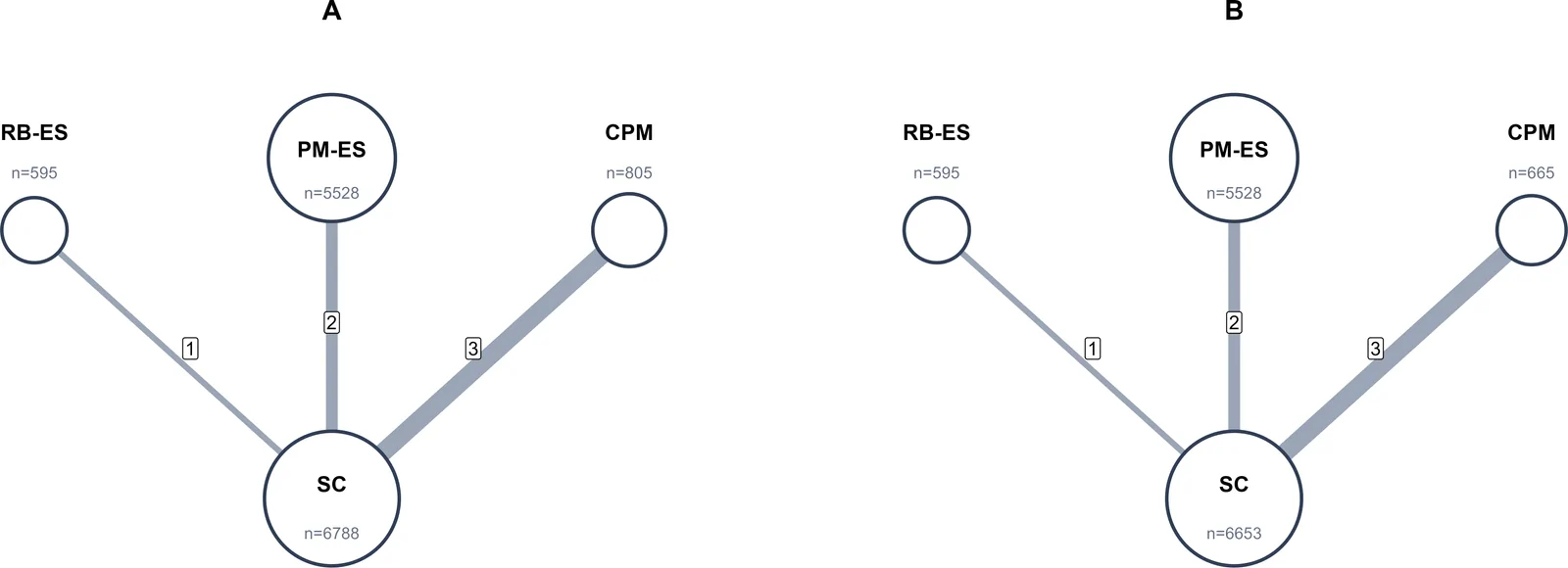
Evidence-network geometry for the primary Bayesian network meta-analyses. (A) In-hospital or up-to-30-day mortality network. (B) Unplanned or emergent intensive care unit (ICU) transfer network. Node size is proportional to the square root of the number of analysis units allocated to each intervention node. Edge labels indicate the number of direct trials. Both networks were star-shaped and contained no direct active-vs-active comparisons. CPM: continuous physiologic monitoring; PM-ES: predictive model–based electronic surveillance; RB-ES: rule-based electronic surveillance; SC: standard care.

The primary mortality and ICU-transfer networks were star-shaped, with local SC as the only common comparator (Figure 3). Each active-vs-standard-care estimate was supported entirely by its corresponding direct comparison.

All comparisons among active surveillance strategies exclusively relied on indirect evidence. For each active-vs-active comparison, half of the contribution arose from each of the 2 direct comparisons linking the active strategies through SC [26] (Figure S3 in Multimedia Appendix 1).

No head-to-head randomized trial has directly compared RB-ES, PM-ES, and CPM.

### Model Convergence and Heterogeneity

All primary and sensitivity models showed satisfactory convergence. The maximum R-hat across all analyses was 1.003, and the minimum effective sample size exceeded 7200. Trace plots showed stable mixing across chains. A small number of isolated Geweke flags were observed, but they were not accompanied by elevated R-hat values, low effective sample sizes, or visible nonconvergence (Figure S1 in Multimedia Appendix 1).

For the strict primary mortality analysis, the posterior median between-study heterogeneity SD was 0.275 (95% credible interval [CrI] 0.016‐0.859). For the strict ICU-transfer analysis, the posterior median between-study heterogeneity SD was 0.162 (95% CrI 0.007‐0.747), indicating substantial uncertainty in the magnitude of heterogeneity.

### Mortality

Six trials with 13,716 analyzed observations contributed to the primary analysis of in-hospital or up-to-30-day mortality [30,32,33,36-38].

**Figure 4.**
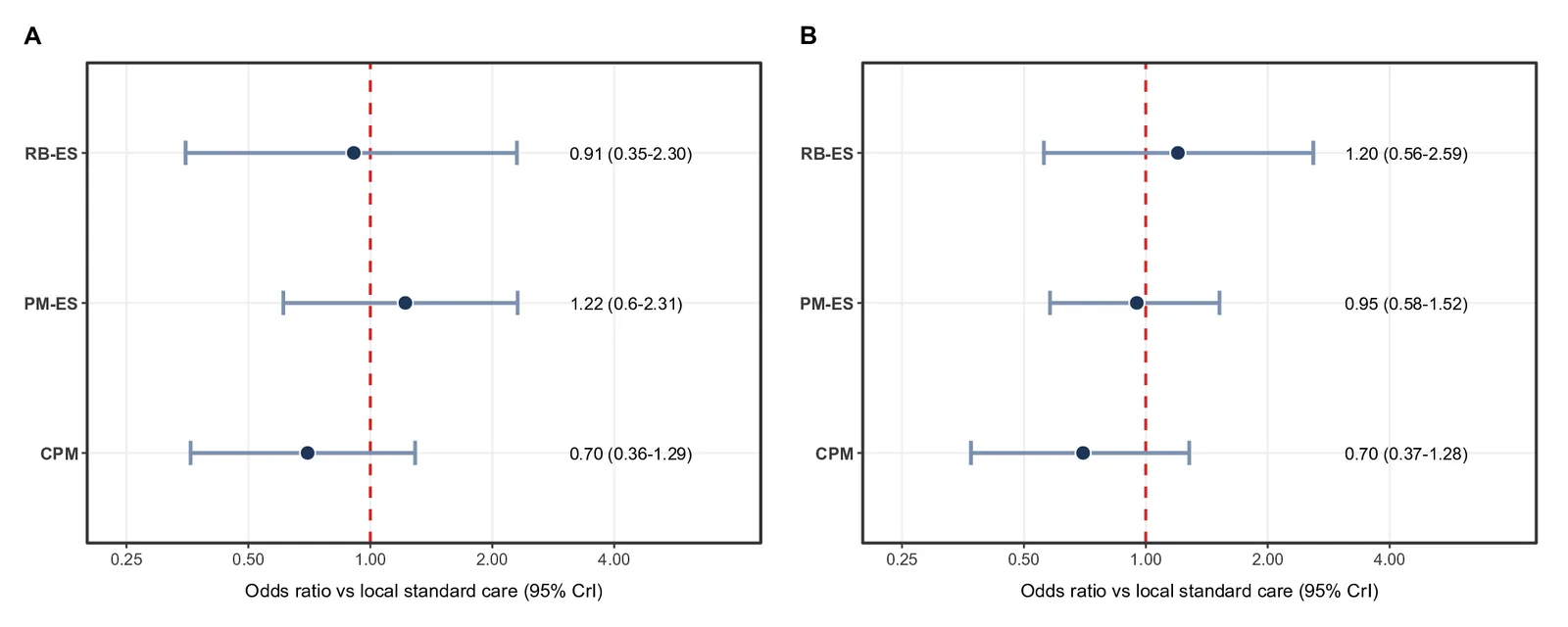
Bayesian random-effects network meta-analysis of the primary outcomes. (A) In-hospital mortality or up-to-30-day mortality. (B) Unplanned or emergent intensive care unit (ICU) transfer. Values are posterior median odds ratios with 95% credible intervals (CrIs) for each surveillance strategy compared with local standard care. No comparison had a CrI that excluded the null value. CPM: continuous physiologic monitoring; PM-ES: predictive model–based electronic surveillance; RB-ES: rule-based electronic surveillance.

No surveillance strategy showed clear evidence of lower mortality compared with local SC (Figure 4A). The OR was 0.91 (95% CrI 0.35‐2.30) for RB-ES, 1.22 (95% CrI 0.61‐2.31) for PM-ES, and 0.70 (95% CrI 0.36‐1.29) for CPM. All CrIs included clinically important benefit and harm.

All active-vs-active comparisons were also imprecise. CPM compared with PM-ES had an OR of 0.57 (95% CrI 0.23‐1.45), CPM compared with RB-ES had an OR of 0.77 (95% CrI 0.24‐2.36), and PM-ES compared with RB-ES had an OR of 1.34 (95% CrI 0.41‐4.11).

An extended follow-up sensitivity analysis added SCREEN, which reported in-hospital deaths occurring within 90 days [31]. The corresponding estimates were 0.86 (95% CrI 0.53‐1.41) for RB-ES, 1.23 (95% CrI 0.69‐2.06) for PM-ES, and 0.70 (95% CrI 0.40‐1.19) for CPM. The inclusion of SCREEN increased the precision of the RB-ES estimate but did not establish a clear class-level mortality benefit.

The results remained directionally consistent across zero-event, study-exclusion, and heterogeneity-prior sensitivity analyses (Figure S2 in Multimedia Appendix 1).

### Unplanned or Emergent ICU Transfer

Six trials, with 13,441 analyzed observations, contributed to the strict ICU-transfer network [30,32,33,35-37].

No surveillance strategy showed a clear effect compared with SC (Figure 4B). The ORs were 1.20 (95% CrI 0.56‐2.59) for RB-ES, 0.95 (95% CrI 0.58‐1.52) for PM-ES, and 0.70 (95% CrI 0.37‐1.28) for CPM.

All active-vs-active comparisons were similarly imprecise. CPM, compared with RB-ES, had an OR of 0.59 (95% CrI 0.22‐1.54); CPM, compared with PM-ES, had an OR of 0.74 (95% CrI 0.34‐1.56); and PM-ES, compared with RB-ES, had an OR of 0.79 (95% CrI 0.32‐1.94).

Broader ICU-admission definitions, inclusion of zero-event trials, and alternative heterogeneity priors did not materially change the findings (Figure S2 in Multimedia Appendix 1). Because ICU transfer may reflect either clinical deterioration or appropriate escalation of care, interventions were not ranked for this outcome.

### Confirmed Cardiac Arrest or Cardiopulmonary Resuscitation

Three expanded rule-based surveillance-response trials reported confirmed cardiac arrest or cardiopulmonary resuscitation [17,40,41].

The pooled OR was 0.94 (95% CI 0.77‐1.14), providing no clear evidence of benefit (Figure S4A in Multimedia Appendix 1). Statistical heterogeneity was not detected (*I*^2^=0%), although its estimate remained imprecise because only 3 trials were available. Code Blue activation, rapid response activation, and cardiac arrest team calls were reported separately.

### Hospital LOS

The core CPM LOS analysis included 3 trials [35,36,38].

The ratio of mean hospital LOS was 0.91 (95% CI 0.76‐1.09), providing no clear evidence that CPM shortened hospitalization (Figure S4B in Multimedia Appendix 1).

An expanded sensitivity analysis of 7 clinically heterogeneous CPM trials yielded a ratio of means (RoM) of 0.86 (95% CI 0.74‐0.99), with substantial heterogeneity (*I*^2^=69.4%). This analysis was considered exploratory because it additionally incorporated passive monitoring, a multicomponent intervention, a cluster trial without cluster-adjusted continuous estimates, and a stroke-specific monitoring unit [47,49-51].

The core PM-ES LOS analysis included 2 trials [32,33]. PM-ES showed no clear effect on LOS in the core analysis (RoM 0.99, 95% CI 0.47‐2.08) or the expanded analysis (RoM 0.98, 95% CI 0.81‐1.19; Figure S4C in Multimedia Appendix 1).

The corrected adjusted incidence rate ratio for the mixed acute care and ICU CONCERN trial was 0.96 (95% CI 0.93‐0.99) and was reported separately because a non-ICU estimate was unavailable [23,45].

### Treatment Activation

The arrival-to-antibiotic or triage-to-antibiotic analysis included 2 trials [43,44].

The random-effects estimate based on reconstructed arithmetic means was imprecise (RoM 0.89, 95% CI 0.51‐1.56; Figure S4D in Multimedia Appendix 1). This result was interpreted cautiously because both trials reported skewed time data, the Kijpaisalratana trial did not report a cluster-adjusted continuous-time estimate, and the Tarabichi intervention combined the alert with pharmacist notification.

Two CPM feasibility trials examined time from suspected deterioration or sepsis identification to antibiotic administration [35,49]. The RoM was 0.78 (95% CI 0.03‐21.83), providing no interpretable evidence of benefit or harm (Figure S4E in Multimedia Appendix 1).

### TSA

Exploratory, retrospective, trial sequential analyses of direct CPM comparisons were inconclusive (Figure S5 in Multimedia Appendix 1).

For mortality, 927 participants represented 11.2% of the required information size of 8262. The pooled RR was 0.98, and the cumulative *Z* curve did not cross a monitoring boundary.

For ICU transfer, 1177 participants represented 5.2% of the required information size of 22,597. The pooled RR was 0.80, and the cumulative *Z* curve did not cross a monitoring boundary.

Because the estimated τ² was 0 in both analyses, fixed-effect and random-effects cumulative *Z* statistics were identical. TSA-adjusted CIs were extremely wide and did not establish benefit, harm, or futility.

### Risk of Bias Due to Missing Evidence

Six trials contributed to each primary network. The descriptive comparison-adjusted funnel plots were sparse and could not be interpreted reliably (Figure S6 in Multimedia Appendix 1). Therefore, formal asymmetry tests and trim-and-fill analyses were not performed.

Across-studies bias was judged to raise some concerns for all primary comparisons because of the small evidence base, the selective availability of patient-centered outcomes from pilot and feasibility trials, and the absence of any additional completed unpublished eligible trials. Publication bias could neither be confirmed nor excluded.

### Confidence in the Evidence

Confidence in all primary network comparisons was rated very low (Multimedia Appendix 3).

The principal reasons were serious imprecision, within-study bias, variation in target populations, and surveillance-response pathways, and exclusive reliance on indirect evidence for all active-vs-active comparisons.

Incoherence could not be evaluated statistically because neither of the primary networks contained a closed loop.

## Discussion

### Principal Findings

This systematic review and Bayesian NMA did not establish a reliable clinical-effectiveness hierarchy among RB-ES, PM-ES, and CPM. None of the 3 surveillance classes showed clear evidence of reducing short-term mortality or altering unplanned or emergent ICU transfers compared with local SC.

CPM produced directionally favorable point estimates for both primary outcomes, but the CrIs remained compatible with a clinically important benefit, no important difference, and clinically important harm. The estimates for rule-based and predictive model–based surveillance were similarly uncertain. Consequently, these results should not be interpreted as evidence that any surveillance class is ineffective; rather, the available randomized evidence is insufficiently precise to confirm or exclude meaningful clinical effects.

Expanded rule-based surveillance-response systems did not clearly reduce confirmed cardiac arrest or cardiopulmonary resuscitation. Core analyses also found no clear class-level effect of CPM or predictive model–based surveillance on hospital LOS. A favorable LOS estimate emerged only in an expanded continuous-monitoring analysis that incorporated passive monitoring, a multicomponent decision-support intervention, a cluster trial without a cluster-adjusted continuous estimate, and a stroke-specific monitoring unit. Therefore, this result was considered hypothesis-generating rather than confirmatory.

The contribution matrices identified a fundamental limitation of the comparative evidence base: no head-to-head randomized trial directly compared RB-ES, PM-ES, and CPM. Every comparison between active surveillance strategies entirely depended on indirect evidence through local SC. This structure substantially limits the certainty of any comparative ranking.

### Comparison With Prior Work and Interpretation

Intervention classification materially shaped the evidence network. Therefore, we classified systems according to the principal randomized contrast, linked multiple reports to their underlying independent trials, and excluded procedural monitoring, treatment-guidance technologies, and disease-specific multicomponent decision support when the independent effect of deterioration surveillance could not be isolated. These restrictions reduced the apparent precision of the evidence base but improved clinical coherence and reduced the risk of generating misleading indirect comparisons from nonexchangeable intervention nodes. The resulting network did not support a class-level advantage for rule-based, predictive model–based, or continuous-monitoring strategies.

After publications were linked to independent trials, procedural and treatment-guidance technologies were excluded, adjusted cluster-trial estimates were prioritized, and the intervention nodes were defined according to their principal randomized function, the apparent rule-based advantage was no longer present. This methodological change was necessary because NMA can produce numerically precise but clinically misleading comparisons when interventions assigned to the same node do not represent exchangeable treatment strategies.

Previous evidence syntheses have highlighted important weaknesses in the development and validation of EWSs, including inconsistent outcome definitions, inadequate handling of missing data, and limited prospective evaluation [4]. Reviews of machine learning–based systems have similarly found that retrospective predictive performance substantially exceeds the amount of evidence demonstrating prospective clinical benefit [5]. Our results extend these findings by showing that randomized effectiveness evidence remains insufficient not only for individual systems but also for establishing a comparative hierarchy among surveillance classes.

The findings illustrate why predictive accuracy should not be equated with clinical effectiveness. Predictive model–based interventions differed in whether their risk information was actively pushed to clinicians or displayed passively, whether alerts were accompanied by an explicit response protocol, and whether clinicians trusted or acted on the output. A model can identify high-risk patients accurately but fail to improve outcomes when it does not alter clinical decisions, responsibility for action is unclear, or the information arrives after clinicians have already recognized the problem.

CPM is also a complex intervention rather than a sensor alone. Continuous data must be technically reliable, transformed into clinically meaningful information, communicated to an appropriate clinician, and followed by a timely and effective response. Trials in which data were passively available through a separate dashboard may therefore evaluate a substantially different intervention compared with systems that transmit filtered alerts directly to responsible nursing or medical staff. The effect of continuous monitoring may be attenuated by alarm fatigue, inadequate staffing, incomplete device adherence, or the absence of a protocol linking specific abnormalities to clinical action [6].

The findings are also consistent with implementation science frameworks emphasizing that adoption, scale-up, and sustainability depend on the interaction between the technology, its users, the organization, and the surrounding care pathway rather than on technical performance alone [3]. However, the present review did not directly compare interpretability or workflow integration as randomized intervention components. It would therefore be inappropriate to conclude that either factor is definitively more important than predictive complexity.

ICU transfer requires particularly cautious interpretation. A reduction in ICU transfer may reflect the prevention of deterioration or avoidance of unnecessary critical care, whereas an increase may reflect earlier recognition and appropriate escalation. In the CONCERN trial, for example, a greater number of unanticipated ICU transfers occurred alongside a lower adjusted instantaneous risk of death, illustrating that ICU transfer cannot be treated as a uniformly undesirable outcome [45]. ICU-transfer results should therefore be interpreted together with mortality, cardiac arrest, organ support, and measures of delayed or appropriate escalation [2].

### Clinical and Research Implications

Hospitals should not select a surveillance technology solely according to whether the underlying algorithm is rule-based, statistically predictive, or machine-learned. The current randomized evidence does not support a class-level purchasing or implementation hierarchy. Directionally favorable estimates for a technology class should not be treated as evidence of superiority when their intervals remain compatible with important benefits and harms.

Technology assessment should instead consider the complete surveillance-response pathway, including data quality, acquisition frequency, threshold selection, alert burden, presentation format, interoperability with the electronic health record, accountability for response, staffing, escalation protocols, and compatibility with existing workflow. Surveillance technologies that use the same algorithm may produce different effects when implemented through different response pathways, whereas technologies using different algorithms may produce similar effects when their clinical workflows are equivalent.

Future effectiveness trials should directly compare active surveillance strategies while standardizing background monitoring and clinical response pathways. Such trials should distinguish appropriate from delayed ICU transfer, prespecify harmonized patient-centered outcomes, account for cluster randomization and calendar effects, and report unintended consequences alongside potential benefits.

Implementation and human-factors outcomes should be evaluated alongside mortality and morbidity. These outcomes should include alert burden, alert acknowledgment, time to bedside assessment, adherence to the assigned surveillance strategy, clinician response, workload, reasons for nonresponse, and the extent to which alerts change diagnostic or treatment decisions. Effectiveness-implementation hybrid designs may be useful when both clinical outcome evaluation and implementation mechanisms are considered essential [57].

Future reports should provide cluster-adjusted estimates and sufficient information to distinguish unique participants from admissions and encounters. Primary reports, protocols, statistical analysis plans, secondary analyses, and corrections should be explicitly linked to prevent duplicate contributions to evidence syntheses.

### Strengths and Limitations

This review has several strengths. Publications were linked to their underlying independent trials, duplicate populations were identified, and intervention nodes were defined according to their principal randomized functions. Study-level effects adjusted for cluster and stepped-wedge designs were prioritized, and hazard ratios, RRs, ORs, and incidence rate ratios were not treated as interchangeable without an explicit harmonization strategy.

Primary and sensitivity analyses were separated according to follow-up duration, outcome definition, clinical setting, intervention scope, background monitoring, and surveillance purpose. Procedural monitoring and monitoring used principally for treatment guidance were not combined with general deterioration surveillance. Continuous outcomes with different clinical anchors were not forced into a single standardized mean-difference network.

Confidence in the network evidence was assessed using CINeMA, study and direct-comparison contributions were quantified, and TSA was not used as a substitute for certainty assessment. Publication and selective outcome-reporting concerns were assessed through registry and protocol linkage and were reported transparently rather than dismissed on the basis of underpowered funnel-plot tests.

Several limitations remain. Few trials contributed to each primary node, and many were pilot or feasibility studies. Both primary networks were star-shaped, so all comparisons among active surveillance strategies entirely relied on indirect evidence, and incoherence could not be evaluated statistically. The absence of a detected inconsistency signal should therefore not be inferred.

Important differences remained in clinical settings, baseline risk, target conditions, enrollment strategies, alert presentations, background monitoring, and response pathways. These differences limit the transitivity assumption, even after stricter node definitions, and explain why all active-vs-active comparisons were rated as very low confidence.

Some adjusted RRs required conversion to ORs using reported control-group risks. Several continuous outcomes required the estimation of arithmetic means and SDs from medians and IQRs. These converted estimates were reserved for exploratory or sensitivity analyses. One study’s event counts were reconstructed from rounded percentages and examined in an exclusion sensitivity analysis.

The strict mortality definition combined in-hospital mortality and mortality reported within 30 days. Although this was more clinically coherent than the original mixed time horizon, differences remained in whether death occurring after discharge was captured. SCREEN reported only in-hospital deaths within 90 days and was therefore included solely in an extended follow-up sensitivity analysis [31].

Publication bias and selective outcome availability could not be excluded. Each primary network contained only 6 trials, precluding reliable statistical assessment of funnel-plot asymmetry [28]. Several pilot or feasibility trials emphasized physiologic, implementation, or usability outcomes without reporting complete patient-centered effectiveness outcomes, and no additional completed unpublished eligible trial was identified.

Finally, aggregate study-level data prevented the evaluation of whether treatment effects differed according to individual patient risk, actual surveillance exposure, alert acknowledgment, or the fidelity of the clinical response. Study-level classification may therefore conceal meaningful variation among individual technologies and implementation contexts.

### Conclusions

Current randomized evidence is insufficient to identify a clinically superior surveillance technology for non-ICU hospital settings. RB-ES, PM-ES, and CPM all had uncertain effects on mortality and unplanned ICU transfers.

Algorithm class alone is therefore an inadequate basis for selecting a surveillance strategy. Future evaluations should treat data acquisition, risk detection, alert communication, workflow integration, and clinical response as components of a single surveillance-response intervention and should directly compare complete pathways using harmonized patient-centered outcomes.

## Supplementary material

10.2196/98205 Multimedia Appendix 1 Supplementary methods, reporting checklists, trial characteristics, risk-of-bias and certainty assessments, additional statistical analyses, and reproducibility information.

10.2196/98205 Multimedia Appendix 2 Characteristics, revised classification, and synthesis role of included independent trials.

10.2196/98205 Multimedia Appendix 3 Summary of findings and confidence in the primary network estimates.

10.2196/98205 Checklist 1 PRISMA 2020 checklist.

## Abbreviations

CINeMA: Confidence in Network Meta-Analysis
CPM: continuous physiologic monitoring
CrI: credible interval
EWS: early warning score
ICU: intensive care unit
JAGS: Just Another Gibbs Sampler
LOS: length of stay
NMA: network meta-analysis
OR: odds ratio
PM-ES: predictive model–based electronic surveillance
PRISMA: Preferred Reporting Items for Systematic Reviews and Meta-Analyses
PRISMA-S: Preferred Reporting Items for Systematic Reviews and Meta-Analyses literature Search Extension
PROSPERO: International Prospective Register of Systematic Reviews
RB-ES: rule-based electronic surveillance
RoM: ratio of means
RR: risk ratio
SC: standard care
TSA: trial sequential analysis
WHO: World Health Organization
