# Supplementary material for: Clinical Surveillance Technologies in Nonintensive Care Unit Hospital Settings: Systematic Review and Bayesian Network Meta-Analysis of Randomized Trials
Source: J Med Internet Res. 2026 Sep 24;28:e98205. doi: 10.2196/98205 (PMC13606342; doi:10.2196/98205)
Supplement: Supplementary file 2 [file jmir-v28-e98205-s001.docx]

**Multimedia Appendix 1**

**Complete Search Strategies and Search Log**

**Search Summary**

The electronic database searches were conducted from inception through February 28, 2026. Targeted supplementary surveillance was subsequently conducted from March 1 through July 22, 2026 to identify newly published reports, corrections, secondary reports, and records associated with completed registered trials. No language restrictions were applied.

| **Source** | **Platform** | **Coverage** | **Search Date** | **Records** |
| --- | --- | --- | --- | --- |
| PubMed | NCBI | Inception to February 28, 2026 | February 28, 2026 | 486 |
| Embase | Embase.com, Elsevier | Inception to February 28, 2026 | February 28, 2026 | 453 |
| CENTRAL | Cochrane Library | Inception to February 28, 2026 | February 28, 2026 | 372 |
| Web of Science Core Collection | Clarivate | Inception to February 28, 2026 | February 28, 2026 | 334 |
| Total database records | — | — | — | 1645 |
| Targeted supplementary surveillance | Trial registries, citation tracking, and known-item searching | March 1 to July 22, 2026 | July 22, 2026 | 14 reports |

Trial registries were used to identify ongoing or completed trials and to link protocols, registrations, statistical analysis plans, corrections, and secondary reports to their underlying trials. No additional completed unpublished eligible trial was identified.

**MEDLINE via PubMed**

#1

(

"Hospital Rapid Response Team"[Mesh]

OR "Decision Support Systems, Clinical"[Mesh]

OR "Monitoring, Physiologic"[Mesh]

OR "Vital Signs"[Mesh]

OR "Artificial Intelligence"[Mesh]

OR "Machine Learning"[Mesh]

)

#2

(

"early warning score*"[Title/Abstract]

OR "early warning system*"[Title/Abstract]

OR EWS[Title/Abstract]

OR MEWS[Title/Abstract]

OR NEWS[Title/Abstract]

OR NEWS2[Title/Abstract]

OR qSOFA[Title/Abstract]

OR "track and trigger"[Title/Abstract]

OR "track-and-trigger"[Title/Abstract]

OR "rapid response system*"[Title/Abstract]

OR "rapid response team*"[Title/Abstract]

OR "medical emergency team*"[Title/Abstract]

OR "critical care outreach"[Title/Abstract]

)

#3

(

(

"clinical deterioration"[Title/Abstract]

OR "patient deterioration"[Title/Abstract]

OR "deteriorating patient*"[Title/Abstract]

OR sepsis[Title/Abstract]

OR "cardiac arrest"[Title/Abstract]

OR "ICU transfer"[Title/Abstract]

)

AND

(

alert*[Title/Abstract]

OR trigger*[Title/Abstract]

OR surveill*[Title/Abstract]

OR screen*[Title/Abstract]

OR detect*[Title/Abstract]

OR predict*[Title/Abstract]

)

)

#4

(

(

"machine learning"[Title/Abstract]

OR "artificial intelligence"[Title/Abstract]

OR "predictive model*"[Title/Abstract]

OR "prediction model*"[Title/Abstract]

OR "risk model*"[Title/Abstract]

OR "risk score*"[Title/Abstract]

OR algorithm*[Title/Abstract]

)

AND

(

alert*[Title/Abstract]

OR warning[Title/Abstract]

OR deteriorat*[Title/Abstract]

OR surveill*[Title/Abstract]

OR sepsis[Title/Abstract]

)

)

#5

(

"continuous monitoring"[Title/Abstract]

OR "continuous vital sign*"[Title/Abstract]

OR "continuous physiologic*"[Title/Abstract]

OR "continuous physiological*"[Title/Abstract]

OR "wireless monitoring"[Title/Abstract]

OR "wearable monitoring"[Title/Abstract]

OR "remote monitoring"[Title/Abstract]

OR "patient-worn monitor*"[Title/Abstract]

OR

(

(

continuous[Title/Abstract]

OR wireless[Title/Abstract]

OR wearable*[Title/Abstract]

OR remote[Title/Abstract]

OR automated[Title/Abstract]

OR "real time"[Title/Abstract]

OR realtime[Title/Abstract]

)

AND

(

"vital sign*"[Title/Abstract]

OR physiologic*[Title/Abstract]

OR physiological[Title/Abstract]

)

)

)

#6

(

"clinical decision support"[Title/Abstract]

OR "electronic alert*"[Title/Abstract]

OR "automated alert*"[Title/Abstract]

OR "real-time alert*"[Title/Abstract]

OR "realtime alert*"[Title/Abstract]

OR "electronic surveillance"[Title/Abstract]

)

#7

#1 OR #2 OR #3 OR #4 OR #5 OR #6

#8

(

"Inpatients"[Mesh]

OR "Hospital Units"[Mesh]

OR inpatient*[Title/Abstract]

OR hospitalized[Title/Abstract]

OR hospitalised[Title/Abstract]

OR ward*[Title/Abstract]

OR "general care"[Title/Abstract]

OR "medical surgical"[Title/Abstract]

OR "medical-surgical"[Title/Abstract]

OR "acute care"[Title/Abstract]

OR "acute admission unit*"[Title/Abstract]

OR "acute medical unit*"[Title/Abstract]

OR "step-down unit*"[Title/Abstract]

OR "step down unit*"[Title/Abstract]

OR "emergency department*"[Title/Abstract]

)

#9

(

randomized controlled trial[Publication Type]

OR controlled clinical trial[Publication Type]

OR random*[Title/Abstract]

OR "stepped wedge"[Title/Abstract]

OR "stepped-wedge"[Title/Abstract]

OR (cluster*[Title/Abstract] AND random*[Title/Abstract])

OR crossover[Title/Abstract]

OR "cross-over"[Title/Abstract]

OR "controlled trial"[Title/Abstract]

OR pragmatic[Title/Abstract]

)

NOT

(

animals[MeSH Terms]

NOT humans[MeSH Terms]

)

#10

#7 AND #8 AND #9

**Embase via Embase.com**

#1

'early warning score'/exp

OR 'rapid response team'/exp

OR 'clinical decision support system'/exp

OR 'patient monitoring'/exp

OR 'vital sign'/exp

OR 'artificial intelligence'/exp

OR 'machine learning'/exp

#2

(

'early warning score*'

OR 'early warning system*'

OR EWS

OR MEWS

OR NEWS

OR NEWS2

OR qSOFA

OR 'track and trigger'

OR 'track-and-trigger'

OR 'rapid response system*'

OR 'rapid response team*'

OR 'medical emergency team*'

OR 'critical care outreach'

):ti,ab,kw

#3

(

(

'clinical deterioration'

OR 'patient deterioration'

OR 'deteriorating patient*'

OR sepsis

OR 'cardiac arrest'

OR 'ICU transfer'

)

NEAR/5

(

alert*

OR trigger*

OR surveill*

OR screen*

OR detect*

OR predict*

)

):ti,ab,kw

#4

(

(

'machine learning'

OR 'artificial intelligence'

OR 'predictive model*'

OR 'prediction model*'

OR 'risk model*'

OR 'risk score*'

OR algorithm*

)

NEAR/5

(

alert*

OR warning

OR deteriorat*

OR surveill*

OR sepsis

)

):ti,ab,kw

#5

(

'continuous monitoring'

OR 'continuous vital sign*'

OR 'continuous physiologic*'

OR 'continuous physiological*'

OR 'wireless monitoring'

OR 'wearable monitoring'

OR 'remote monitoring'

OR 'patient-worn monitor*'

OR

(

(

continuous

OR wireless

OR wearable*

OR remote

OR automated

OR 'real time'

OR realtime

)

NEAR/3

(

monitor*

OR 'vital sign*'

OR physiologic*

OR physiological

)

)

):ti,ab,kw

#6

(

'clinical decision support'

OR 'electronic alert*'

OR 'automated alert*'

OR 'real-time alert*'

OR 'realtime alert*'

OR 'electronic surveillance'

):ti,ab,kw

#7

#1 OR #2 OR #3 OR #4 OR #5 OR #6

#8

'hospital patient'/exp

OR 'hospital ward'/exp

OR 'emergency ward'/exp

OR

(

inpatient*

OR hospitalized

OR hospitalised

OR ward*

OR 'general care'

OR 'medical surgical'

OR 'medical-surgical'

OR 'acute care'

OR 'acute admission unit*'

OR 'acute medical unit*'

OR 'step-down unit*'

OR 'step down unit*'

OR 'emergency department*'

):ti,ab,kw

#9

'randomized controlled trial'/exp

OR 'controlled clinical trial'/exp

OR

(

random*

OR 'stepped wedge'

OR 'stepped-wedge'

OR 'cluster random*'

OR crossover

OR 'cross-over'

OR 'controlled trial'

OR pragmatic

):ti,ab,kw

#10

#7 AND #8 AND #9

#11

#10 NOT ('animal'/exp NOT 'human'/exp)

**CENTRAL via the Cochrane Library**

#1 MeSH descriptor: [Monitoring, Physiologic] explode all trees

#2 MeSH descriptor: [Vital Signs] explode all trees

#3 MeSH descriptor: [Decision Support Systems, Clinical] explode all trees

#4 MeSH descriptor: [Hospital Rapid Response Team] explode all trees

#5 MeSH descriptor: [Artificial Intelligence] explode all trees

#6

(

"early warning score*"

OR "early warning system*"

OR EWS

OR MEWS

OR NEWS

OR NEWS2

OR qSOFA

OR "track and trigger"

OR "track-and-trigger"

OR "rapid response system*"

OR "rapid response team*"

OR "medical emergency team*"

OR "critical care outreach"

):ti,ab,kw

#7

(

(

"clinical deterioration"

OR "patient deterioration"

OR "deteriorating patient*"

OR sepsis

)

NEAR/5

(

alert*

OR trigger*

OR surveill*

OR screen*

OR detect*

OR predict*

)

):ti,ab,kw

#8

(

(

continuous

OR wireless

OR wearable*

OR remote

OR automated

OR "real time"

OR realtime

)

NEAR/3

(

monitor*

OR "vital sign*"

OR physiologic*

OR physiological

)

):ti,ab,kw

#9

(

(

"machine learning"

OR "artificial intelligence"

OR "predictive model*"

OR "prediction model*"

OR "risk model*"

OR "risk score*"

OR algorithm*

)

NEAR/5

(

alert*

OR warning

OR deteriorat*

OR surveill*

OR sepsis

)

):ti,ab,kw

#10

#1 OR #2 OR #3 OR #4 OR #5 OR #6 OR #7 OR #8 OR #9

#11

(

inpatient*

OR hospitalized

OR hospitalised

OR ward*

OR "general care"

OR "medical surgical"

OR "medical-surgical"

OR "acute care"

OR "acute admission unit*"

OR "acute medical unit*"

OR "step-down unit*"

OR "emergency department*"

):ti,ab,kw

#12

#10 AND #11

**Web of Science Core Collection**

#1 TS=(

"early warning score*"

OR "early warning system*"

OR EWS

OR MEWS

OR NEWS

OR NEWS2

OR qSOFA

OR "track and trigger"

OR "track-and-trigger"

OR "rapid response system*"

OR "rapid response team*"

OR "medical emergency team*"

OR "critical care outreach"

OR "clinical decision support"

OR "electronic alert*"

OR "automated alert*"

OR "real-time alert*"

OR "electronic surveillance"

)

#2 TS=(

(

"clinical deterioration"

OR "patient deterioration"

OR "deteriorating patient*"

OR sepsis

)

NEAR/5

(

alert*

OR trigger*

OR surveill*

OR screen*

OR detect*

OR predict*

)

)

#3 TS=(

(

continuous

OR wireless

OR wearable*

OR remote

OR automated

OR "real time"

OR realtime

)

NEAR/3

(

monitor*

OR "vital sign*"

OR physiologic*

OR physiological

)

)

#4 TS=(

(

"machine learning"

OR "artificial intelligence"

OR "predictive model*"

OR "prediction model*"

OR "risk model*"

OR "risk score*"

OR algorithm*

)

NEAR/5

(

alert*

OR warning

OR deteriorat*

OR surveill*

OR sepsis

)

)

#5 #1 OR #2 OR #3 OR #4

#6 TS=(

inpatient*

OR hospitali?ed

OR ward*

OR "general care"

OR "medical surgical"

OR "medical-surgical"

OR "acute care"

OR "acute admission unit*"

OR "acute medical unit*"

OR "step-down unit*"

OR "emergency department*"

)

#7 TS=(

random*

OR "controlled trial"

OR "stepped wedge"

OR "stepped-wedge"

OR "cluster trial"

OR crossover

OR "cross-over"

OR pragmatic

)

#8 #5 AND #6 AND #7

**Supplementary Search Methods**

The following searches were undertaken in ClinicalTrials.gov and the World Health Organization International Clinical Trials Registry Platform:

"early warning system" hospital ward

"clinical deterioration" alert hospital

"continuous vital sign monitoring" ward

"machine learning" deterioration alert hospital

"sepsis alert" randomized

"rapid response system" randomized hospital

The reference lists of all included trials and relevant systematic reviews were examined. Forward citation searches were performed in Google Scholar. Fourteen additional reports were identified through backward and forward citation tracking and updated supplementary searching.

**Multimedia Appendix 2**

**PRISMA-NMA Reporting Checklist**

| **Item** | **Reporting Requirement** | **Location in Revised Manuscript** |
| --- | --- | --- |
| 1 | Identify the report as a systematic review incorporating a network meta-analysis | Title |
| 2 | Provide a structured summary | Abstract |
| 3 | Describe rationale in context of existing evidence | Introduction |
| 4 | Provide explicit objectives | Introduction, final paragraph |
| 5 | State protocol and registration information | Methods, Study Design and Reporting |
| 6 | Describe eligibility criteria and report characteristics | Methods, Eligibility Criteria |
| 7 | Describe information sources and last search date | Methods, Information Sources and Search Strategy |
| 8 | Present complete search strategies | Multimedia Appendix 1 |
| 9 | Describe study-selection process | Methods, Study Selection and Data Extraction |
| 10 | Describe data-collection and verification processes | Methods, Study Selection and Data Extraction |
| 11 | Define outcomes, nodes, and data items | Methods, Outcome Definitions; Intervention Classification |
| 12 | Describe methods for linking multiple reports of the same trial | Methods, Trial-Report Linkage and Data Units |
| 13 | Describe within-study risk-of-bias methods | Methods, Risk of Bias |
| 14 | Describe effect measures | Methods, Effect-Measure Selection and Harmonization |
| 15 | Describe network geometry | Methods, Network Geometry and Contribution Analysis |
| 16 | Describe transitivity assessment | Methods, Transitivity Assessment |
| 17 | Describe statistical synthesis and model assumptions | Methods, Bayesian Network Meta-Analysis |
| 18 | Describe heterogeneity assumptions and priors | Methods, Bayesian Network Meta-Analysis |
| 19 | Describe inconsistency or incoherence assessment | Methods, Network Geometry and Contribution Analysis |
| 20 | Describe ranking methods or explain their omission | Methods, Bayesian Network Meta-Analysis |
| 21 | Describe sensitivity and secondary analyses | Methods, Sensitivity Analyses; Secondary Meta-Analyses |
| 22 | Describe missing-evidence or publication-bias assessment | Methods, Risk of Bias Due to Missing Evidence |
| 23 | Describe certainty-of-evidence assessment | Methods, Certainty of Evidence |
| 24 | Report study selection | Results, Study Selection; Figure 1 |
| 25 | Report trial characteristics | Results, Study Characteristics; Table 1 |
| 26 | Present network structure and direct evidence | Results, Network Geometry; Figure 3 |
| 27 | Present study and direct-comparison contributions | Results, Network Geometry; Supplementary Figure S3 |
| 28 | Present risk-of-bias results | Results, Risk of Bias; Figure 2 |
| 29 | Present network estimates with uncertainty | Results, Mortality and ICU Transfer; Figure 4 |
| 30 | Present heterogeneity and convergence | Results, Model Convergence and Heterogeneity |
| 31 | Present sensitivity analyses | Results; Supplementary Figure S2 |
| 32 | Present certainty judgments | Results, Confidence in the Evidence; Table 2 |
| 33 | Discuss limitations of evidence and review methods | Discussion, Strengths and Limitations |
| 34 | Provide implications for practice and research | Discussion, Clinical and Research Implications |
| 35 | Report funding, conflicts, data, and code availability | End Matter |

**Multimedia Appendix 3**

**PRISMA-S Reporting Checklist**

| **Item** | **Search Reporting Requirement** | **Reported Information** |
| --- | --- | --- |
| 1 | Database name | MEDLINE, Embase, CENTRAL, Web of Science Core Collection |
| 2 | Platform | PubMed/NCBI; Embase.com/Elsevier; Cochrane Library; Clarivate |
| 3 | Trial registries | ClinicalTrials.gov and WHO ICTRP |
| 4 | Additional information sources | Backward and forward citation tracking and Google Scholar |
| 5 | Contact with study authors | Used when clarification or unavailable data were sought; non-ICU subgroup data from Rossetti were not available |
| 6 | Complete strategies | Presented in Multimedia Appendix 1 |
| 7 | Limits and restrictions | No language restrictions; inception through February 28, 2026 |
| 8 | Search dates | February 28, 2026 |
| 9 | Search peer review | Search strategy developed and reviewed with medical librarians at Xiangya Hospital |
| 10 | Search update | Targeted supplementary surveillance, including trial-registry follow-up, backward and forward citation tracking, and known-item searches for newly available reports of registered trials, continued through July 22, 2026. |
| 11 | Search yield | PubMed 486; Embase 453; CENTRAL 372; Web of Science 334 |
| 12 | Deduplication | EndNote 21 (Clarivate Analytics, Philadelphia, PA), followed by manual review |
| 13 | Citation-management software | EndNote 21 (Clarivate Analytics, Philadelphia, PA) |
| 14 | Screening software | Microsoft Excel spreadsheet (Microsoft 365) |
| 15 | Full-text retrieval | All 109 reports sought for full-text assessment were retrieved |
| 16 | Reproducibility materials | Search strategies, trial-report linkage provided in appendices |

**Multimedia Appendix 4**

**Trial-Report Linkage and Included Trial Identifiers**

**Table S1. Important Multiple Reports and Trial Linkages**

| **Trial ID** | **Registration** | **Primary Effectiveness Report** | **Linked Reports** | **Decision** |
| --- | --- | --- | --- | --- |
| ePNa | NCT03358342 | Dean et al, 2022 | Carr et al, 2024 | Same trial; excluded from surveillance network |
| Haegdorens | NCT01949025 | Haegdorens et al, 2018 | Haegdorens et al, 2019 | Same trial; 2019 report used for implementation context |
| Weenk | NCT02933307 | Weenk et al, 2019 | Weenk et al, 2020 | Same trial; excluded because no eligible patient-centered effectiveness outcome |
| SCREEN | NCT04078594 | Arabi et al, 2025 | Protocol and statistical analysis plan | Same trial; protocol and SAP used for reporting-bias assessment |
| CONCERN | NCT03911687 | Rossetti et al, 2025 | 2026 correction | Same trial; corrected LOS estimate used |
| COSMOS | NCT05280574 | Anusic et al, 2024 | 2026 corrigendum | Same trial; excluded from patient-centered effectiveness syntheses |
| I-EWS | NCT03690128 | Nielsen et al, 2022 | Protocol/implementation reports | Same trial; within-node comparison |
| Kijpaisalratana | TCTR20230120001 | Kijpaisalratana et al, 2024 | Thai registry record | Retrospectively registered |
| Kant | NCT05181111 | Kant et al, 2024 | Protocol/registry | Passive CPM sensitivity analysis |
| CoMET | NCT04359641 | Keim-Malpass et al, 2026 | Protocol | Strict PM-ES evidence set |
| Khanna | NCT04574908 | Khanna et al, 2026 | Online supplement | Strict CPM evidence set |
| Mølgaard | NCT04640415 | Mølgaard et al, 2025 | Online supplement | Strict CPM evidence set |
| Lin AI-ECG | NCT05118035 | Lin et al, 2024 | Registry record | Mixed ED-inpatient sensitivity analysis |

**Table S2. Complete Titles and DOI Numbers of the 28 Included Trials**

| **No.** | **Study** | **Complete Trial-Report Title** | **DOI** |
| --- | --- | --- | --- |
| 1 | Sulter 2003 | Admitting Acute Ischemic Stroke Patients to a Stroke Care Monitoring Unit Versus a Conventional Stroke Unit: A Randomized Pilot Study | 10.1161/01.STR.0000048148.09143.6C |
| 2 | Priestley 2004 | Introducing Critical Care Outreach: a ward-randomised trial of phased introduction in a general hospital | 10.1007/s00134-004-2268-7 |
| 3 | Hillman 2005 | Introduction of the medical emergency team (MET) system: a cluster-randomised controlled trial | 10.1016/S0140-6736(05)66733-5 |
| 4 | Watkinson 2006 | A randomised controlled trial of the effect of continuous electronic physiological monitoring on the adverse event rate in high risk medical and surgical patients | 10.1111/j.1365-2044.2006.04818.x |
| 5 | Langhorne 2010 | Very Early Rehabilitation or Intensive Telemetry after Stroke: A Pilot Randomised Trial | 10.1159/000278931 |
| 6 | Bailey 2013 | A Trial of a Real-Time Alert for Clinical Deterioration in Patients Hospitalized on General Medical Wards | 10.1002/jhm.2009 |
| 7 | Kollef 2014 | A Randomized Trial of Real-Time Automated Clinical Deterioration Alerts Sent to a Rapid Response Team | 10.1002/jhm.2193 |
| 8 | Jeddian 2016 | A mixed methods evaluation of a critical care outreach service in a middle-income country: a stepped wedge cluster randomised trial and nested qualitative study | 10.1016/j.jcrc.2016.07.018 |
| 9 | Petersen 2016 | Frequency of early warning score assessment and clinical deterioration in hospitalized patients: a randomized trial | 10.1016/j.resuscitation.2016.02.003 |
| 10 | Haegdorens 2018 | The introduction of a rapid response system in acute hospitals: a pragmatic stepped wedge cluster randomised controlled trial | 10.1016/j.resuscitation.2018.04.018 |
| 11 | Downey 2018 | Continuous Versus Intermittent Vital Signs Monitoring Using a Wearable, Wireless Patch in Patients Admitted to Surgical Wards: Pilot Cluster Randomized Controlled Trial | 10.2196/10802 |
| 12 | Wong 2018 | Impact of Electronic Versus Paper Vital Sign Observations on Length of Stay in Trauma Patients: Stepped-Wedge, Cluster Randomized Controlled Trial | 10.2196/10221 |
| 13 | Paul 2019 | Vital sign monitoring with continuous pulse oximetry and wireless clinical notification after surgery (the VIGILANCE pilot study)—a randomized controlled pilot trial | 10.1186/s40814-019-0415-8 |
| 14 | Kyriacos 2019 | Testing effectiveness of the revised Cape Town modified early warning and SBAR systems: a pilot pragmatic parallel group randomised controlled trial | 10.1186/s13063-019-3916-0 |
| 15 | Downing 2019 | Electronic health record-based clinical decision support alert for severe sepsis: a randomised evaluation | 10.1136/bmjqs-2018-008765 |
| 16 | Downey 2020 | Trial of remote continuous versus intermittent NEWS monitoring after major surgery (TRaCINg): a feasibility randomised controlled trial | 10.1186/s40814-020-00709-8 |
| 17 | Skraastad 2020 | Postoperative quality and safety using Efficacy Safety Score and a wireless patient monitoring system at the ward: A randomised controlled study | 10.1111/aas.13492 |
| 18 | Nielsen 2022 | Clinical assessment as a part of an early warning score—a Danish cluster-randomised, multicentre study of an individual early warning score | 10.1016/S2589-7500(22)00067-X |
| 19 | Tarabichi 2022 | Improving Timeliness of Antibiotic Administration Using a Provider and Pharmacist Facing Sepsis Early Warning System in the Emergency Department Setting: A Randomized Controlled Quality Improvement Initiative | 10.1097/CCM.0000000000005267 |
| 20 | Kant 2024 | Enhancing discharge decision-making through continuous monitoring in an acute admission ward: a randomized controlled trial | 10.1007/s11739-024-03582-y |
| 21 | Kijpaisalratana 2024 | Real-time machine learning-assisted sepsis alert enhances the timeliness of antibiotic administration and diagnostic accuracy in emergency department patients with sepsis: a cluster-randomized trial | 10.1007/s11739-024-03535-5 |
| 22 | Posthuma 2024 | Surveillance of high-risk early postsurgical patients for real-time detection of complications using wireless monitoring (SHEPHERD study): results of a randomized multicenter stepped wedge cluster trial | 10.3389/fmed.2023.1295499 |
| 23 | Lin 2024 | AI-enabled electrocardiography alert intervention and all-cause mortality: a pragmatic randomized clinical trial | 10.1038/s41591-024-02961-4 |
| 24 | Rossetti 2025 | Real-time surveillance system for patient deterioration: a pragmatic cluster-randomized controlled trial | 10.1038/s41591-025-03609-7 |
| 25 | Arabi/SCREEN 2025 | Electronic Sepsis Screening Among Patients Admitted to Hospital Wards: A Stepped-Wedge Cluster Randomized Trial | 10.1001/jama.2024.25982 |
| 26 | Mølgaard 2025 | Continuous Vital Sign Monitoring at the Surgical Ward for Improved Outcomes After Major Noncardiac Surgery: A Randomized Clinical Trial | 10.1213/ANE.0000000000007606 |
| 27 | Keim-Malpass 2026 | A randomized controlled trial of artificial intelligence-based analytics for clinical deterioration | 10.1038/s41598-026-39051-z |
| 28 | Khanna 2026 | Continuous vs Intermittent Postoperative Vital Sign Monitoring: A Cluster Randomized Crossover Trial | 10.1001/jamanetworkopen.2026.3290 |

**Note: SCREEN was published online in December 2024 and assigned to a 2025 journal issue. The SHEPHERD report was published online in January 2024 with an electronic collection year of 2023.**

**Multimedia Appendix 5**

**Intervention Node Assignment, Transitivity, and Full-Text Exclusions**

**Table S3. Node Assignment and Synthesis Role**

| **Study** | **Primary Randomized Function** | **Assigned Category** | **Background Monitoring** | **Alert/Display** | **Analysis Role** |
| --- | --- | --- | --- | --- | --- |
| Sulter 2003 | Continuous stroke-unit physiologic monitoring | CPM | Conventional stroke care | Continuous display | Stroke sensitivity |
| Priestley 2004 | PAR score plus critical care outreach | Expanded RB response | Local usual care | Triggered referral | Sensitivity |
| Hillman 2005 | MET criteria plus medical emergency team | Expanded RB response | Local usual care | Staff-activated criteria | Sensitivity |
| Watkinson 2006 | Continuous multiparameter monitoring | CPM | Intermittent ward monitoring | Bedside alarms/display | Strict evidence |
| Langhorne 2010 | Automated physiologic monitoring after stroke | CPM | 4-hourly monitoring | Automated display | Stroke sensitivity |
| Bailey 2013 | Logistic-regression deterioration alert | PM-ES | Routine ward care | Alert to nurse manager | Methodological sensitivity |
| Kollef 2014 | Predictive alert linked to rapid response team | PM-ES | Routine ward care | Active RRT alert | Strict evidence |
| Jeddian 2016 | Single-parameter trigger plus outreach team | Expanded RB response | Local usual care | Ward referral criteria | Sensitivity |
| Petersen 2016 | EWS frequency comparison | Within-node RB | EWS | No node contrast | Narrative |
| Haegdorens 2018 | NEWS, SBAR, and pragmatic response pathway | Expanded RB response | Local usual care | Structured escalation | Sensitivity |
| Downey 2018 | Continuous wearable monitoring added to NEWS | CPM | NEWS | Wireless alert | Expanded sensitivity |
| Wong 2018 | Electronic versus paper EWS recording | Within-node RB | Same EWS | Electronic implementation | Narrative |
| Paul 2019 | Continuous pulse oximetry with notification | CPM | Intermittent monitoring | Wireless alert | Strict/zero-event sensitivity |
| Kyriacos 2019 | Cape Town MEWS plus SBAR | Expanded RB | Standard chart | Structured score/escalation | Narrative |
| Downing 2019 | Deterministic severe-sepsis EHR alert | RB-ES | Usual care | Active page to clinical team | Strict evidence |
| Downey 2020 | Continuous wearable monitoring plus NEWS | CPM | NEWS | Wireless alert | Strict/sensitivity |
| Skraastad 2020 | Wireless monitoring plus ESS | CPM, multicomponent | Standard postoperative care | Bedside display | Expanded sensitivity |
| Nielsen 2022 | Individual EWS versus NEWS | Within-node RB | NEWS-like comparator | EWS workflow | Narrative |
| Tarabichi 2022 | Predictive sepsis alert plus pharmacist notification | PM-ES | Routine ED care | Active EHR/pharmacist alert | ED sensitivity |
| Kant 2024 | Passive continuous wearable monitoring | CPM | EWS | Separate dashboard, no alarm | Passive sensitivity |
| Kijpaisalratana 2024 | Random-forest sepsis alert | PM-ES | Human sepsis-alert process | Display alert | ED sensitivity |
| Posthuma 2024 | Wireless continuous monitoring | CPM | Standard postoperative care | Wireless monitoring | Narrative |
| Lin 2024 | AI-ECG mortality-risk alert | PM-ES | Usual care | Physician alert | Mixed-setting sensitivity |
| Rossetti 2025 | ML model of nursing-surveillance metadata | PM-ES | Usual care | EHR display | Mixed-setting sensitivity |
| SCREEN 2025 | qSOFA deterministic electronic screening | RB-ES | Usual care | Nurse/physician alert | Extended sensitivity |
| Mølgaard 2025 | Continuous monitoring with smartphone alert | CPM | NEWS | Filtered smartphone alert | Strict mortality/secondary LOS |
| Keim-Malpass 2026 | Predictive risk-trajectory display | PM-ES | Usual care | Passive visual display | Strict evidence |
| Khanna 2026 | Continuous physiologic display and alert | CPM | Intermittent monitoring | Active alert | Strict evidence |

**Table S4. Transitivity Assessment Across Strict Nodes**

| **Effect Modifier** | **RB-ES** | **PM-ES** | **CPM** | **Implication** |
| --- | --- | --- | --- | --- |
| Main settings | General medical/surgical wards | General medical/cardiac wards | High-risk and postoperative wards | Setting imbalance may modify effect |
| Target event | Severe sepsis/general deterioration | General deterioration or imminent adverse event | Physiologic instability | Target conditions differ |
| Data frequency | Intermittent EHR data | Intermittent/streamed EHR data | Continuous/near-continuous physiologic data | Detection opportunity differs |
| Alert format | Active alert/page | Active alert or passive display | Display or active alarm | Actionability differs |
| Background monitoring | Routine intermittent monitoring | Routine ward care | Intermittent monitoring or NEWS | Comparator intensity differs |
| Response pathway | Nurse/physician assessment | RRT, clinical team, or no mandated response | Nurse-led or clinician-led response | Response fidelity differs |
| Baseline risk | Sepsis-alert-positive or general ward populations | Alert-positive/general cardiac ward populations | Selected high-risk/postoperative populations | Baseline risk unevenly distributed |
| Follow-up | In-hospital/30 days; 90 days in SCREEN sensitivity | In-hospital/21 days | In-hospital/30 days | Time horizon differs |
| Overall implication | — | — | — | Active-versus-active estimates interpreted as exploratory and downgraded for indirectness |

**Table S5. Reports Excluded After Full-Text Assessment That Could Reasonably Be Considered Eligible**

| **Report** | **Exclusion Reason** |
| --- | --- |
| Miner et al, The Utility of the Bispectral Index in Procedural Sedation With Propofol in the Emergency Department | Procedural sedation monitoring |
| Deitch et al, Does End Tidal CO₂ Monitoring During Emergency Department Procedural Sedation and Analgesia With Propofol Decrease Hypoxic Events? | Procedural sedation monitoring |
| Huh et al, Comparison of Intermittent and Continuous ScvO₂ Monitoring | Treatment-guidance monitoring; both groups actively monitored |
| Hou et al, Cardiac Output Monitoring Managing Intravenous Therapy | NICOM-guided fluid treatment |
| Kuan et al, Emergency Department Management of Sepsis Patients | NICOM/passive-leg-raise fluid treatment |
| Dean et al, Real-Time Pneumonia Clinical Decision Support | Disease-specific multicomponent CDS; nonrandomized rollout order |
| Carr et al, ICU Utilization After ePNa Implementation | Secondary report of Dean trial; disease-specific CDS |
| Nadaf et al, Traditional vs AI-Assisted Triage Systems | Triage rather than repeated deterioration surveillance |
| van Dam et al, MARS-ED | One-time 31-day prognostic risk presentation |
| Weenk et al, 2019 | Device measurement and high-MEWS detection only |
| Weenk et al, 2020 | User experience and implementation outcomes only |
| Anusic et al/COSMOS | Physiologic abnormality duration and nursing response outcomes |
| Kyriacos et al, 2015 | Documentation and nursing behavior outcomes only |
| Bellomo et al, 2012 | Nonrandomized controlled implementation study |
| Brown et al, 2014 | Nonrandomized controlled implementation study |
| Sawyer et al, 2011 | Prospective nonrandomized alert implementation |
| Manaktala et al, 2017 | Nonrandomized surveillance implementation |
| Escobar et al | Nonrandomized predictive EWS implementation |
| Verma et al | Nonrandomized controlled cohort |
| Gallo et al, 2024 | Regression-discontinuity study |
| Eddahchouri et al, 2022 | Before-and-after study |
| Rowland et al, 2024 | Propensity-matched observational study |
| Leenen et al, 2024 | Before-and-after study |
| Micek et al | Retrospective alert analysis |
| Yadav et al | Observational continuous-monitoring cohort |
| Jonkers et al | Device agreement or feasibility without effectiveness outcome |

**Full-Text Exclusion Summary**

| **Category** | **Number** |
| --- | --- |
| Wrong intervention or surveillance purpose | 35 |
| Ineligible study design | 22 |
| Ineligible population or setting | 15 |
| No eligible patient-centered outcome or insufficient data | 9 |
| Total | 81 |

**Multimedia Appendix 6**

**Risk of Bias and CINeMA Certainty Assessments**

**Table S6. RoB 2 Judgments for the Strict Digital-Surveillance Evidence Set**

| **Study** | **Randomization** | **Recruitment in Relation to Clusters** | **Deviations** | **Missing Data** | **Outcome Measurement** | **Selective Reporting** | **Overall** |
| --- | --- | --- | --- | --- | --- | --- | --- |
| Downing 2019 | Some concerns | Not applicable | Low | Low | Low | Some concerns | Some concerns |
| SCREEN 2025 | Some concerns | Low | Some concerns | Low | Low | Low | Some concerns |
| Kollef 2014 | Low | Not applicable | Low | Low | Low | Low | Low |
| Keim-Malpass 2026 | Low | Low | High | High | Low | Low | High |
| Watkinson 2006 | Low | Not applicable | Some concerns | Low | Low | Low | Some concerns |
| Paul 2019 | Low | Not applicable | High | Low | Low | Some concerns | High |
| Downey 2020 | Low | Not applicable | High | Some concerns | Low | Some concerns | High |
| Mølgaard 2025 | Low | Not applicable | Some concerns | Low | Low | Some concerns | Some concerns |
| Khanna 2026 | Some concerns | Some concerns | Some concerns | Some concerns | Low | Some concerns | Some concerns |

**Table S7. Manual CINeMA Assessment for Mortality**

| **Comparison** | **Effect, OR (95% CrI)** | **Within-Study Bias** | **Reporting Bias** | **Indirectness** | **Imprecision** | **Heterogeneity** | **Incoherence** | **Confidence** |
| --- | --- | --- | --- | --- | --- | --- | --- | --- |
| RB-ES vs SC | 0.91 (0.35-2.30) | Some concerns | Some concerns | Some concerns | Major concerns | No additional concerns | Not assessable | Very low |
| PM-ES vs SC | 1.22 (0.61-2.31) | Major concerns | Some concerns | Some concerns | Major concerns | No additional concerns | Not assessable | Very low |
| CPM vs SC | 0.70 (0.36-1.29) | Some concerns | Some concerns | Some concerns | Major concerns | No additional concerns | Not assessable | Very low |
| PM-ES vs RB-ES | 1.34 (0.41-4.11) | Major concerns | Some concerns | Major concerns | Major concerns | No additional concerns | Not assessable | Very low |
| CPM vs RB-ES | 0.77 (0.24-2.36) | Some concerns | Some concerns | Major concerns | Major concerns | No additional concerns | Not assessable | Very low |
| CPM vs PM-ES | 0.57 (0.23-1.45) | Major concerns | Some concerns | Major concerns | Major concerns | No additional concerns | Not assessable | Very low |

**Table S8. Manual CINeMA Assessment for ICU Transfer**

| **Comparison** | **Effect, OR (95% CrI)** | **Within-Study Bias** | **Reporting Bias** | **Indirectness** | **Imprecision** | **Heterogeneity** | **Incoherence** | **Confidence** |
| --- | --- | --- | --- | --- | --- | --- | --- | --- |
| RB-ES vs SC | 1.20 (0.56-2.59) | Some concerns | Some concerns | Some concerns | Major concerns | No additional concerns | Not assessable | Very low |
| PM-ES vs SC | 0.95 (0.58-1.52) | Major concerns | Some concerns | Some concerns | Major concerns | No additional concerns | Not assessable | Very low |
| CPM vs SC | 0.70 (0.37-1.28) | Some concerns | Some concerns | Some concerns | Major concerns | No additional concerns | Not assessable | Very low |
| PM-ES vs RB-ES | 0.79 (0.32-1.94) | Major concerns | Some concerns | Major concerns | Major concerns | No additional concerns | Not assessable | Very low |
| CPM vs RB-ES | 0.59 (0.22-1.54) | Some concerns | Some concerns | Major concerns | Major concerns | No additional concerns | Not assessable | Very low |
| CPM vs PM-ES | 0.74 (0.34-1.56) | Major concerns | Some concerns | Major concerns | Major concerns | No additional concerns | Not assessable | Very low |

**Table S9. Across-Studies Bias Assessment**

| **Outcome** | **Comparison** | **Direct Trials** | **Formal Asymmetry Test** | **Judgment** | **Rationale** |
| --- | --- | --- | --- | --- | --- |
| Mortality | RB-ES vs SC | 1 | Not performed | Some concerns | Single direct trial; mortality not primary efficacy outcome |
| Mortality | PM-ES vs SC | 2 | Not performed | Some concerns | Sparse evidence; 1 high-risk trial |
| Mortality | CPM vs SC | 3 | Not performed | Some concerns | Pilot/feasibility evidence and incomplete outcome availability |
| ICU transfer | RB-ES vs SC | 1 | Not performed | Some concerns | Single direct trial |
| ICU transfer | PM-ES vs SC | 2 | Not performed | Some concerns | Sparse evidence and heterogeneous response pathways |
| ICU transfer | CPM vs SC | 3 | Not performed | Some concerns | Small trials, rare events, and pilot evidence |

**Multimedia Appendix 7**

**Additional Statistical Results**

**Table S10. Bayesian League Table for Mortality**

Each cell is the posterior median OR for the column-defining intervention versus the row-defining comparator.

| **Comparator \ Intervention** | **SC** | **RB-ES** | **PM-ES** | **CPM** |
| --- | --- | --- | --- | --- |
| SC | — | 0.91 (0.35-2.30) | 1.22 (0.61-2.31) | 0.70 (0.36-1.29) |
| RB-ES | 1.10 (0.44-2.82) | — | 1.34 (0.41-4.11) | 0.77 (0.24-2.36) |
| PM-ES | 0.82 (0.43-1.65) | 0.74 (0.24-2.43) | — | 0.57 (0.23-1.45) |
| CPM | 1.44 (0.77-2.78) | 1.30 (0.42-4.17) | 1.74 (0.69-4.40) | — |

**Table S11. Bayesian League Table for ICU Transfer**

| **Comparator \ Intervention** | **SC** | **RB-ES** | **PM-ES** | **CPM** |
| --- | --- | --- | --- | --- |
| SC | — | 1.20 (0.56-2.59) | 0.95 (0.58-1.52) | 0.70 (0.37-1.28) |
| RB-ES | 0.84 (0.39-1.80) | — | 0.79 (0.32-1.94) | 0.59 (0.22-1.54) |
| PM-ES | 1.06 (0.66-1.71) | 1.26 (0.52-3.09) | — | 0.74 (0.34-1.56) |
| CPM | 1.42 (0.78-2.70) | 1.70 (0.65-4.60) | 1.34 (0.64-2.96) | — |

**Table S12. Mortality Sensitivity Analyses**

| **Analysis** | **RB-ES vs SC** | **PM-ES vs SC** | **CPM vs SC** |
| --- | --- | --- | --- |
| Strict primary mortality | 0.91 (0.35-2.30) | 1.22 (0.61-2.31) | 0.70 (0.36-1.29) |
| Extended follow-up including SCREEN | 0.86 (0.53-1.41) | 1.23 (0.69-2.06) | 0.70 (0.40-1.19) |
| Extended follow-up plus zero-event study | 0.86 (0.53-1.41) | 1.22 (0.69-2.07) | 0.72 (0.43-1.23) |
| Extended follow-up excluding Downing | 0.85 (0.36-1.94) | 1.22 (0.61-2.31) | 0.70 (0.37-1.30) |
| Heterogeneity prior scale 0.2 | 0.85 (0.64-1.17) | 1.23 (0.79-1.88) | 0.70 (0.45-1.08) |
| Heterogeneity prior scale 1.0 | 0.86 (0.48-1.59) | 1.22 (0.62-2.26) | 0.70 (0.37-1.28) |

**Table S13. ICU-Transfer Sensitivity Analyses**

| **Analysis** | **RB-ES vs SC** | **PM-ES vs SC** | **CPM vs SC** |
| --- | --- | --- | --- |
| Strict unplanned/emergent transfer | 1.20 (0.56-2.59) | 0.95 (0.58-1.52) | 0.70 (0.37-1.28) |
| Broader ICU-admission definition | 1.05 (0.76-1.56) | 0.95 (0.66-1.37) | 0.70 (0.42-1.17) |
| Broader definition plus zero-event trial | 1.05 (0.76-1.54) | 0.95 (0.67-1.37) | 0.73 (0.44-1.22) |
| Heterogeneity prior scale 0.2 | 1.04 (0.83-1.37) | 0.95 (0.73-1.25) | 0.70 (0.43-1.13) |
| Heterogeneity prior scale 1.0 | 1.05 (0.72-1.66) | 0.95 (0.63-1.46) | 0.70 (0.40-1.20) |

**Table S14. Secondary Outcome Meta-Analyses**

| **Outcome** | **Trials** | **Random-Effects Estimate** | **95% CI** | **I²** |
| --- | --- | --- | --- | --- |
| Confirmed cardiac arrest/CPR, expanded RB systems | 3 | OR 0.94 | 0.77-1.14 | 0% |
| CPM and LOS, core | 3 | RoM 0.91 | 0.76-1.09 | 7.5% |
| CPM and LOS, expanded | 7 | RoM 0.86 | 0.74-0.99 | 69.4% |
| PM-ES and LOS, core | 2 | RoM 0.99 | 0.47-2.08 | 46.4% |
| PM-ES and LOS, expanded | 4 | RoM 0.98 | 0.81-1.19 | 63.1% |
| PM-ES arrival/triage to antibiotics | 2 | RoM 0.89 | 0.51-1.56 | 30.8% |
| CPM deterioration to antibiotics | 2 | RoM 0.78 | 0.03-21.83 | 24.4% |

**Table S15. Exploratory Trial Sequential Analyses**

| **Outcome** | **Included Trials** | **Direct Trials** | **Accumulated Information** | **Required Information Size** | **Information Fraction** | **Pooled RR** | **Naive P Value** | **Conclusion** |
| --- | --- | --- | --- | --- | --- | --- | --- | --- |
| CPM and mortality | Watkinson 2006; Downey 2020; Mølgaard 2025 | 3 | 927 | 8262 | 11.2% | 0.98 | .9069 | Inconclusive |
| CPM and ICU transfer | Watkinson 2006; Paul 2019; Downey 2020; Mølgaard 2025 | 4 | 1177 | 22,597 | 5.2% | 0.80 | .5074 | Inconclusive |

**Note:Trials were entered sequentially according to publication year. Analyses assumed a 20% relative risk reduction, 2-sided α=.05, 80% power, Lan-DeMets O’Brien-Fleming alpha-spending boundaries, no futility boundary, Mantel-Haenszel weighting, diversity adjustment, and a continuity correction of 0.5 for zero cells.**

**Table S16. Direct-Comparison Contribution Matrix**

| **Network Comparison** | **CPM:SC** | **PM-ES:SC** | **RB-ES:SC** |
| --- | --- | --- | --- |
| CPM vs PM-ES | 50% | 50% | 0% |
| CPM vs RB-ES | 50% | 0% | 50% |
| CPM vs SC | 100% | 0% | 0% |
| PM-ES vs RB-ES | 0% | 50% | 50% |
| PM-ES vs SC | 0% | 100% | 0% |
| RB-ES vs SC | 0% | 0% | 100% |

**Table S17. Primary Trial Reports Added Through Citation Tracking and Targeted Supplementary Surveillance**

| **No.** | **Study** | **Full Report Title** | **DOI** | **Primary Identification Route** | **Role in the Revised Review** |
| --- | --- | --- | --- | --- | --- |
| 1 | Sulter et al, 2003 | Admitting Acute Ischemic Stroke Patients to a Stroke Care Monitoring Unit Versus a Conventional Stroke Unit: A Randomized Pilot Study | 10.1161/01.STR.0000048148.09143.6C | Backward citation tracking of stroke-unit and continuous-monitoring literature | CPM; stroke-specific sensitivity analysis |
| 2 | Priestley et al, 2004 | Introducing Critical Care Outreach: a Ward-Randomised Trial of Phased Introduction in a General Hospital | 10.1007/s00134-004-2268-7 | Backward citation tracking of critical care outreach and rapid-response-system reviews | Expanded rule-based surveillance-response sensitivity analysis |
| 3 | Hillman et al, 2005 | Introduction of the Medical Emergency Team (MET) System: a Cluster-Randomised Controlled Trial | 10.1016/S0140-6736(05)66733-5 | Backward citation tracking of medical emergency team and rapid-response-system reviews | Expanded rule-based surveillance-response sensitivity analysis for cardiac arrest and ICU outcomes |
| 4 | Watkinson et al, 2006 | A Randomised Controlled Trial of the Effect of Continuous Electronic Physiological Monitoring on the Adverse Event Rate in High Risk Medical and Surgical Patients | 10.1111/j.1365-2044.2006.04818.x | Backward citation tracking of continuous physiologic monitoring reviews | Strict CPM primary evidence and exploratory TSA |
| 5 | Langhorne et al, 2010 | Very Early Rehabilitation or Intensive Telemetry After Stroke: A Pilot Randomised Trial | 10.1159/000278931 | Backward citation tracking of stroke telemetry and continuous-monitoring literature | CPM; stroke-specific sensitivity analysis |
| 6 | Bailey et al, 2013 | A Trial of a Real-Time Alert for Clinical Deterioration in Patients Hospitalized on General Medical Wards | 10.1002/jhm.2009 | Backward citation tracking of predictive deterioration-alert literature | PM-ES methodological sensitivity analysis |
| 7 | Kollef et al, 2014 | A Randomized Trial of Real-Time Automated Clinical Deterioration Alerts Sent to a Rapid Response Team | 10.1002/jhm.2193 | Forward citation tracking from earlier predictive-alert studies and backward citation tracking of alert-system reviews | Strict PM-ES primary evidence |
| 8 | Jeddian et al, 2016 | Evaluation of a Critical Care Outreach Service in a Middle-Income Country: A Stepped Wedge Cluster Randomized Trial and Nested Qualitative Study | 10.1016/j.jcrc.2016.07.018 | Forward and backward citation tracking of critical care outreach literature | Expanded rule-based surveillance-response sensitivity analysis |
| 9 | Petersen et al, 2016 | Frequency of Early Warning Score Assessment and Clinical Deterioration in Hospitalized Patients: A Randomized Trial | 10.1016/j.resuscitation.2016.02.003 | Backward citation tracking of early warning score and track-and-trigger reviews | Within-node rule-based monitoring comparison; narrative synthesis |
| 10 | Haegdorens et al, 2018 | The Introduction of a Rapid Response System in Acute Hospitals: A Pragmatic Stepped Wedge Cluster Randomised Controlled Trial | 10.1016/j.resuscitation.2018.04.018 | Forward and backward citation tracking of rapid-response-system trials | Expanded rule-based surveillance-response sensitivity analysis |
| 11 | Downey et al, 2018 | Continuous Versus Intermittent Vital Signs Monitoring Using a Wearable, Wireless Patch in Patients Admitted to Surgical Wards: Pilot Cluster Randomized Controlled Trial | 10.2196/10802 | Backward citation tracking of wearable continuous-monitoring reviews | Expanded CPM sensitivity and treatment-activation analyses |
| 12 | Paul et al, 2019 | Vital Sign Monitoring With Continuous Pulse Oximetry and Wireless Clinical Notification After Surgery (the VIGILANCE Pilot Study)—a Randomized Controlled Pilot Trial | 10.1186/s40814-019-0415-8 | Backward citation tracking of postoperative continuous pulse oximetry and wearable-monitoring literature | Strict CPM evidence; zero-event ICU-transfer sensitivity analysis and exploratory TSA |
| 13 | Downey et al, 2020 | Trial of Remote Continuous Versus Intermittent NEWS Monitoring After Major Surgery (TRaCINg): a Feasibility Randomised Controlled Trial | 10.1186/s40814-020-00709-8 | Forward citation tracking from the earlier Downey wearable-monitoring trial and targeted known-item searching | Strict CPM evidence, length-of-stay analysis, treatment-activation analysis, and exploratory TSA |
| 14 | Khanna et al, 2026 | Continuous vs Intermittent Postoperative Vital Sign Monitoring: A Cluster Randomized Crossover Trial | 10.1001/jamanetworkopen.2026.3290 | Targeted follow-up of ClinicalTrials.gov registration NCT04574908 and known-item surveillance after the database-search cutoff | Strict CPM primary evidence and exploratory TSA |

**Note:** This table presents the primary randomized trial reports added to the final review library through backward or forward citation tracking and targeted supplementary surveillance. Each report was assigned to a single primary identification route after deduplication. Protocols, trial registrations, statistical analysis plans, secondary reports, corrections, and online supplements used only for trial linkage, reporting-bias assessment, or correction of extracted estimates were documented separately and were not counted as independent trials. Khanna et al was published on March 2, 2026, after the February 28, 2026 database-search cutoff, and was identified through targeted follow-up of ClinicalTrials.gov registration NCT04574908. CPM indicates continuous physiologic monitoring; ICU, intensive care unit; PM-ES, predictive model–based electronic surveillance; and TSA, trial sequential analysis.

**Multimedia Appendix 8**

**Protocol Deviations and Reviewer-Requested Analytical Amendments**

| **Original Plan or Manuscript** | **Revised Approach** | **Reason** | **Status** |
| --- | --- | --- | --- |
| Search through February 2026 | Search updated through July 22, 2026 | Major revision and identification of omitted randomized trials | Search update |
| Randomized and high-quality quasi-experimental studies eligible | Randomized, cluster-randomized, crossover, and stepped-wedge randomized trials retained for main review | Increase internal validity and respond to design concerns | Reviewer-requested |
| R-EWS, ML-EWS, CPM, SC nodes | RB-ES, PM-ES, CPM, and SC nodes | Original nodes were not clinically defensible | Reviewer-requested |
| Disease-specific ePNa classified as R-EWS | ePNa excluded from surveillance network | Multicomponent pneumonia CDS and nonrandomized rollout order | Reviewer-requested |
| Procedure, ScvO₂, and NICOM studies included in CPM | Removed | Procedural safety or treatment guidance rather than general surveillance | Reviewer-requested |
| Arm-level binomial models using raw counts | Contrast-based NMA using design-adjusted effects | Avoid unit-of-analysis error in cluster trials | Methodological correction |
| ORs, HRs, and IRRs treated as comparable | Different ratio measures separated; HRs not pooled with ORs | Preserve estimand and time-to-event distinctions | Methodological correction |
| All-cause mortality combined across varying horizons | In-hospital or ≤30-day mortality primary; SCREEN 90-day in-hospital mortality sensitivity | Improve clinical coherence | Reviewer-requested |
| Any ICU admission treated as uniformly adverse | Strict unplanned/emergent ICU transfer primary; broader admissions sensitivity | ICU transfer may represent appropriate escalation | Methodological correction |
| Clinical deterioration composite included hypoxia, sepsis, SOFA deterioration, ICU transfer, and arrest | Constructs analyzed separately | Outcomes were not clinically equivalent | Reviewer-requested |
| LOS pooled using SMD | Ratio of arithmetic means; adjusted IRRs separately | LOS is right-skewed and measured in common time units | Reviewer-requested |
| TTA pooled as a single SMD network | Split by time origin and clinical construct | Different treatment-activation constructs | Reviewer-requested |
| Three MCMC chains and manuscript settings inconsistent with code | Four chains; 20,000 adaptation; 100,000 sampling; thinning 10 | Align manuscript with final analysis | Correction |
| Model selected by DIC | Random-effects model prespecified; fixed effect sensitivity only | Clinical heterogeneity anticipated | Methodological correction |
| Meta-regression by publication year and sample size | Removed | Too few studies for reliable meta-regression | Reviewer-requested |
| Subgroup analyses using old nodes | Removed or replaced with clinically defined sensitivity analyses | Original subgroups were invalid after reclassification | Correction |
| SUCRA rankings emphasized | Removed from clinical conclusions | Sparse star-shaped networks and no active head-to-head evidence | Reviewer-requested |
| Node splitting and no inconsistency reported | Incoherence reported as not assessable | No closed loops in strict networks | Reviewer-requested |
| Egger, Begg, and trim-and-fill | Removed; descriptive funnel plots only | Fewer than 10 studies per network | Methodological correction |
| Trial sequential analysis used to claim firm evidence | Restricted to exploratory retrospective direct CPM comparisons | TSA does not replace certainty assessment | Reviewer-requested |
| No certainty assessment | Manual CINeMA assessment added | Address bias, indirectness, imprecision, heterogeneity, and incoherence | Reviewer-requested |
| “First NMA” claim | Removed | Priority could not be established with sufficient certainty | Reviewer-requested |
| PROSPERO registration described as prospective | Registration claim removed | Identifier could not be independently verified | Transparency correction |
| Cohen κ stated but not reported | Statement removed | Original screening data insufficient for valid retrospective κ | Reviewer-requested |
| Publication bias described as absent | Changed to “could not be reliably assessed” | Sparse networks | Reviewer-requested |

**Multimedia Appendix 9**

**Data Dictionary, Analytical File Inventory, and Reproducibility Information**

**Analysis Environment**

R version 4.4.1

Platform: x86_64-w64-mingw32/x64

Operating system: Windows 11 x64

Primary packages:

gemtc 1.0-2

rjags 4-17

coda 0.19-4.1

netmeta 3.1-1

meta 8.0-2

metafor 4.8-0

RTSA 0.2.2

ggplot2 4.0.3

dplyr 1.2.1

readr 2.1.5

**Core Data Files**

| **File** | **Purpose** |
| --- | --- |
| 04_mortality_effects_primary.csv | Study-level effects for mortality |
| 05_icu_transfer_effects_primary.csv | Study-level effects for ICU transfer |
| 06a_confirmed_cardiac_arrest_REWS.csv | Cardiac arrest/CPR effects |
| 06b_code_blue_RRT_and_other_escalation.csv | Nonpooled escalation outcomes |
| 07a_adjusted_LOS_ratio_estimates.csv | Adjusted LOS ratio measures |
| 08a_antibiotic_process_outcomes.csv | Treatment-activation outcomes |
| Secondary_outcomes_study_level_RoM.csv | Study-level ratios of means |
| RoB2_working_judgments.csv | RoB 2 domain-level judgments |
| CINeMA_mortality_primary.csv | Mortality CINeMA input |
| CINeMA_ICU_primary.csv | ICU-transfer CINeMA input |

**Core Analytical Scripts**

| **Script** | **Purpose** |
| --- | --- |
| NMA_v5_1_contrast_based.R | Primary Bayesian NMA and sensitivity analyses |
| NMA_v5_2_export_diagnostics.R | Convergence and diagnostics export |
| Secondary_outcomes_v5_3.R | Cardiac arrest, LOS, and treatment-time analyses |
| CINeMA_RoB_v5_4.R | CINeMA input and RoB tables |
| Final_contribution_tables_v5_5.R | Study/direct contribution and league tables |
| Final_Figures_From_Frozen_Results.R | Main figures from frozen estimates |
| Final_Supplementary_Figures_From_Frozen_Results.R | Supplementary figures from frozen estimates |
| TSA_v5_2.R | Exploratory retrospective TSA |

**Primary Variable Dictionary**

| **Variable** | **Definition** |
| --- | --- |
| study | Unique trial identifier |
| treat1 | Intervention node |
| treat2 | Comparator node |
| measure | Original effect measure |
| estimate | Original reported adjusted estimate |
| lower, upper | Lower and upper 95% confidence limits |
| event1, n1 | Intervention events and denominator |
| event0, n0 | Comparator events and denominator |
| p0 | Comparator-group event risk |
| OR_used | Odds ratio used in primary NMA |
| logOR | Natural logarithm of odds ratio |
| seLogOR | Standard error of log odds ratio |
| primary_include | Prespecified primary-analysis eligibility |
| strict_definition | Eligibility for strict ICU-transfer definition |
| background_EWS | Whether both randomized groups received an existing EWS |
| rob | CINeMA within-study bias code: 1 low, 2 some concerns, 3 high |
| indirectness | CINeMA indirectness code: 1 low, 2 some concerns, 3 major concerns |

**Reproducibility Statement**

All primary numerical figures were generated from frozen CSV or RDS outputs. The final figure scripts do not rerun the Bayesian models and therefore reproduce the exact posterior medians and credible intervals used in the manuscript.
